# Evolving dispersal ability causes rapid adaptive radiation

**DOI:** 10.1038/s41598-024-66435-w

**Published:** 2024-07-08

**Authors:** Takeshi Yamasaki, Yutaka Kobayashi

**Affiliations:** 1 Yamashina Institute for Ornithology, 115 Konoyama, Abiko, Chiba 270-1145 Japan; 2 School of Economics and Management, Kochi University of Technology, 2-22 Eikokuji-Cho, Kochi, 780-8515 Japan

**Keywords:** Speciation, Adaptive radiation

## Abstract

Despite a long history of research since Darwin, the mechanism underlying rapid adaptive radiation remains poorly understood. All theories constructed to date require special assumptions, so none can comprehensively explain actual cases found in wide-ranging taxonomic groups. Here, we propose a simple theoretical solution to this problem. Namely, we extend the classical archipelago model of adaptive radiation into a more realistic model by adding one assumption, namely, the evolvability of dispersal ability, which is well supported empirically. Our individual-based simulations with evolvable dispersal ability showed that environmental heterogeneity among islands (or island-like habitats) led to an evolutionary decrease in dispersal ability. However, when islands are rather evenly distributed, as is often the case in actual archipelagos where adaptive radiation has been reported, the decline in dispersal ability that began in some island populations was quickly halted by the continuous influx of immigrants from other islands. The process of reduction in dispersal ability in these island populations was resumed almost synchronously when the dispersal ability began to decrease on the final island, which had maintained high dispersal ability and continued to release migrants for the longest duration. Then, a rapid loss of dispersal ability followed in all island populations. In short, the frequent simultaneous evolution of multiple allopatric incipient species was an inevitable consequence of the properties of ordinary archipelagos in our simulations. This study strongly suggests that the seemingly complex process of rapid radiation is driven by a simple mechanism of evolutionary reduction in dispersal ability.

## Introduction

Adaptive radiation is the evolution of remarkably ecologically diverse descendant species from a single ancestral species^1–8^ triggered by access to underutilized resources or ecological opportunity^6,9^. Adaptive radiation often gives rise to many species in a short period of time, but the mechanisms that enable this rapid sequence of speciation are not well understood^10^. Recent genome-scale phylogenetic analyses^11–14^ have further complicated this issue: these analyses have found phylogenetic patterns of multi-branching, the most extreme form of rapid radiation, in a wide variety of taxa. In these cases, the interval between successive speciation events is too short to be statistically distinguishable from zero. Because many loci have already been studied to identify such polytomies, additional data are unlikely to improve the resolution. Polytomies with this property are called ‘hard polytomies’, as opposed to ‘soft polytomies’ where multi-branching implies low resolution due to a lack of data^15^. Hard polytomies have been found in various organisms, including birds^11^, whales^13^, gingers^12^ and legumes^14^. Possible examples of hard polytomy have also been found in Lake Malawi and Lake Tanganyika cichlids^11^.

Most empirical researchers of adaptive radiation have accepted a theory called the archipelago model^2,5,7^ and have considered allopatric speciation, that is, speciation through the prevention of gene flow by physical barriers, as the primary mode of speciation in adaptive radiation (for example, Refs.^2,5,16–18^). However, under this theory, it is difficult to explain rapid radiation and its most extreme form, simultaneous speciation. This is because adaptive radiation through allopatric speciation requires the simultaneous or nearly simultaneous appearance of numerous physical barriers or rapid and transient range expansion of a species across numerous physical barriers^15,18^; both of these ad hoc scenarios, however, seem to be biologically unrealistic.

In contrast, theoretical researchers of adaptive radiation have been trying to explain rapid radiation and simultaneous speciation exclusively using models of nonallopatric speciation (for example, Refs.^10,19–22^). Despite such efforts, however, it remains difficult to explain rapid radiation. In nonallopatric speciation models, divergent natural selection often plays a major role as the driving force of speciation. Under this assumption, the first speciation in radiation is generally expected to produce two daughter species that have the greatest ecological differences. Then, when each of those two daughter species diverges into two granddaughter species, the ecological differences between those granddaughter species must inevitably be smaller than the differences between the daughter species. Likewise, the intensity of divergent selection should decrease as speciation occurs repeatedly, resulting in a rather longer waiting time to the next speciation event. This problem was recently named the ‘paradox of rapid radiation’ by Martin and Richards^10^.

Although it is theoretically known that rapid radiation and simultaneous speciation can occur even under nonallopatric speciation models, this requires the fulfilment of specific conditions. For example, the theory of Gavrilets et al.^19^ not only requires a sudden and abrupt decrease in dispersal ability from unknown causes, but also depends heavily on parameter settings; adaptive radiation hence does not easily occur^20^. The theory of Gavrilets and Vose^21^ depends on a specific speciation mechanism that is applicable to sympatric speciation in phytophagous insects^23^. Bolnick’s theory^22^ is based on adaptive dynamics theory^24^, and as the author himself admits, his parameter settings are unrealistic. Martin and Richards^10^ discussed the possibility of rapid radiation under the transporter hypothesis, the signal complexity hypothesis, the fitness landscape connectivity hypothesis, the diversity begets diversity hypothesis and the flexible stem/plasticity first hypothesis. However, empirical support for the assumptions of these theories, if any, is available in only a few taxa. Therefore, it is unclear to what extent the combination of these specific theories can explain actual cases of rapid radiation observed in a wide range of taxa, including plants^12,14^, molluscs^25^, insects^26^, fish^27,28^, reptiles^27^, birds^11,27^ and mammals^13,27^.

Here, we propose a simple idea to resolve this dilemma: we incorporate the evolvability of dispersal ability into the classical archipelago model of adaptive radiation, which has been repeatedly adopted by empirical researchers. Note that the intensity of physical barriers depends not only on their physical properties, such as the width of straits and the height of mountain ranges, but also on the dispersal ability of the organisms. Most features that significantly influence dispersal ability, such as the wings of birds and insects, fruit spines, and fruit structures providing buoyancy, have a genetic basis. Indeed, while it seems unlikely that multiple physical barriers could emerge simultaneously or alter their physical properties simultaneously, it is possible that local populations of ancestral species evolve to lose dispersal ability simultaneously. Therefore, the assumption of evolvability of dispersal ability, which should be satisfied in most organism groups (for example, see Refs.^29–33^), can substantially improve the biological plausibility of the scenario of rapid radiation through the simultaneous or near-simultaneous establishment of multiple physical barriers.

The modified scenario is outlined as follows. First, ancestral species that were able to invade a small archipelago far from continents must have had a high initial dispersal ability, for example, the ability to fly long distances or to withstand long periods of drift. Second, the ecological opportunity^6,9^ that triggers adaptive radiation implies the existence of many types of underutilized resources, but the relative frequency of such resources usually varies among subareas of the archipelago where adaptive radiation takes place. For example, the Hawaiian, Galapagos and Macaronesian islands, which are well known for large-scale adaptive radiation events, are all composed of both high-altitude islands with substantial rainfall and dry low-altitude islands^34–37^. Divergent natural selection resulting from such environmental heterogeneity, even under strong gene flow due to high dispersal ability of the ancestral species, can cause local adaptation to evolve^38^. Then, once local adaptation is established, individuals tend to adapt to the subarea of their birth. Under these conditions, dispersal increases the number of opportunities for individuals to encounter environments to which they are not adapted, resulting in negative selection pressure on dispersal ability. Therefore, if dispersal ability is an evolvable trait, it should inevitably decrease through natural selection^38–41^. As a result of such a reduction in dispersal ability, previously non-functional physical barriers will be strengthened without changing their physical characteristics, eventually resulting in genetic isolation of subpopulations. Then, the genetic isolation between islands is highly synchronized under ordinary conditions, as our simulations reveal below, resulting in the simultaneous emergence of many incipient species.

In this study, we performed individual-based simulations using the modified archipelagic model, which assumes the evolvability of dispersal ability. The aim was to see if, as outlined above, (1) local adaptation evolves, (2) dispersal ability is reduced and (3) a highly synchronized intensification of physical barriers, that is, simultaneous speciation, occurs.

## Results

Here, we analysed archipelagos consisting of three islands. We assume that each island is locally environmentally homogeneous, but the environments differ between islands. We exclude the trivial case with only a single environment type, in which all islands have the same environmental conditions and hence divergent selection never operates. We found that the choice of two or three environment types did not essentially affect the results (see “Methods”), and thus we present below only the case with three islands with two environment types. We refer to the two environmentally identical islands as islands B and C, and the environmentally unique island as island A. For ease of understanding, we provisionally describe the first two as low-altitude islands and the latter as a high-altitude island. Although the ancestral species may be adapted to either the high- or the low-altitude conditions, we found that this initial condition had no essential effect on the results (see “Methods”). Therefore, it is assumed below that the ancestral species is adapted to the low-altitude islands, islands B and C, following the taxon cycle theory^42^. This theory aims to explain the characteristics of island biota, arguing that ancestral species newly arriving on islands tend to be adapted to low-altitude coastal zones, but will eventually invade higher altitudes.

The first of our predictions, concerning the evolution of local adaptations, was met in almost all simulations conducted with various parameter settings (a total of 133,300 simulations), except when divergent selection was very weak. Despite strong initial gene flow, the high-altitude and low-altitude island populations almost invariably underwent the fixation of different alleles adapted to their respective environments. However, when divergent selection was very weak, such local adaptations did not always evolve. Next, our second prediction, concerning the evolutionary reduction in dispersal ability, was always met in all simulations. When local adaptation was established by divergent selection, the frequency of the primitive alleles promoting dispersal decreased in all island populations, and eventually, for all island pairs, the average number of migrating individuals per local population per generation always fell below one; thus, three allopatric incipient species were judged to arise based on the OMPG (One Migrant Per Generation) rule (see “Methods” and Refs.^43–45^). Meanwhile, even when divergent selection was weak and local adaptation did not evolve, longer simulations inevitably led to the formation of three allopatric incipient species due to the stochastic loss of dispersal alleles. However, the incipient species generated by this process had short lifespans and often fused back into a single species as a result of random drift increasing dispersal ability again.

In contrast, the third prediction on simultaneous speciation was conditionally met, depending on the parameter settings. We arbitrarily, but very conservatively, assumed that simultaneous speciation occurred when the interval between the first and second speciations was 10 generations or less (see “Methods” and Refs.^46,47^). As for the range of parameters we explored, simultaneous speciation as defined above occurred 13,023 times (9.8%), which even included cases where the first and second speciations occurred in exactly the same generation (speciation interval = 0; 648 simulations, 0.5% of the total).

**Figure 1:**
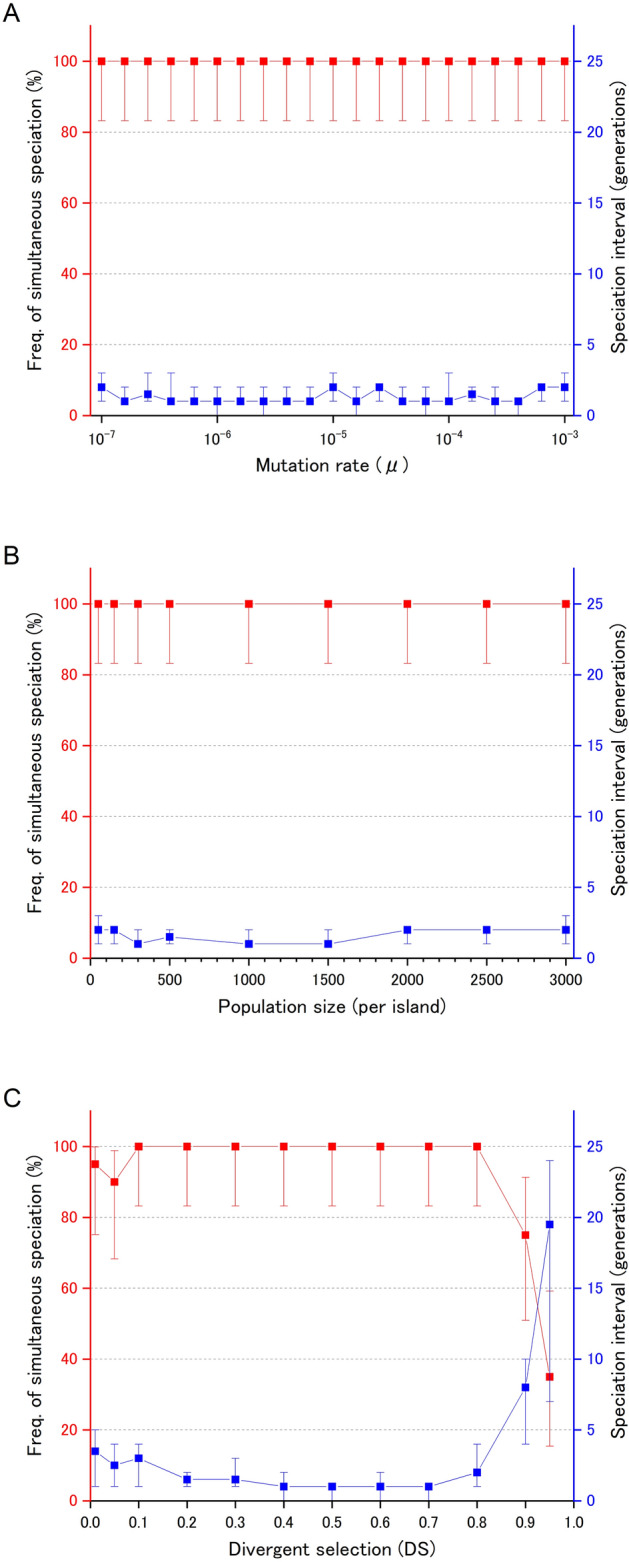
Effect of the mutation rate μ(**A**), the population size (**B**), and the intensity of divergent natural selection DS (**C**) on the frequency of simultaneous speciation (red) and the median speciation interval (blue). Each point is based on 20 simulations run with that setting. Error bars represent 95% confidence intervals.

**Figure 2:**
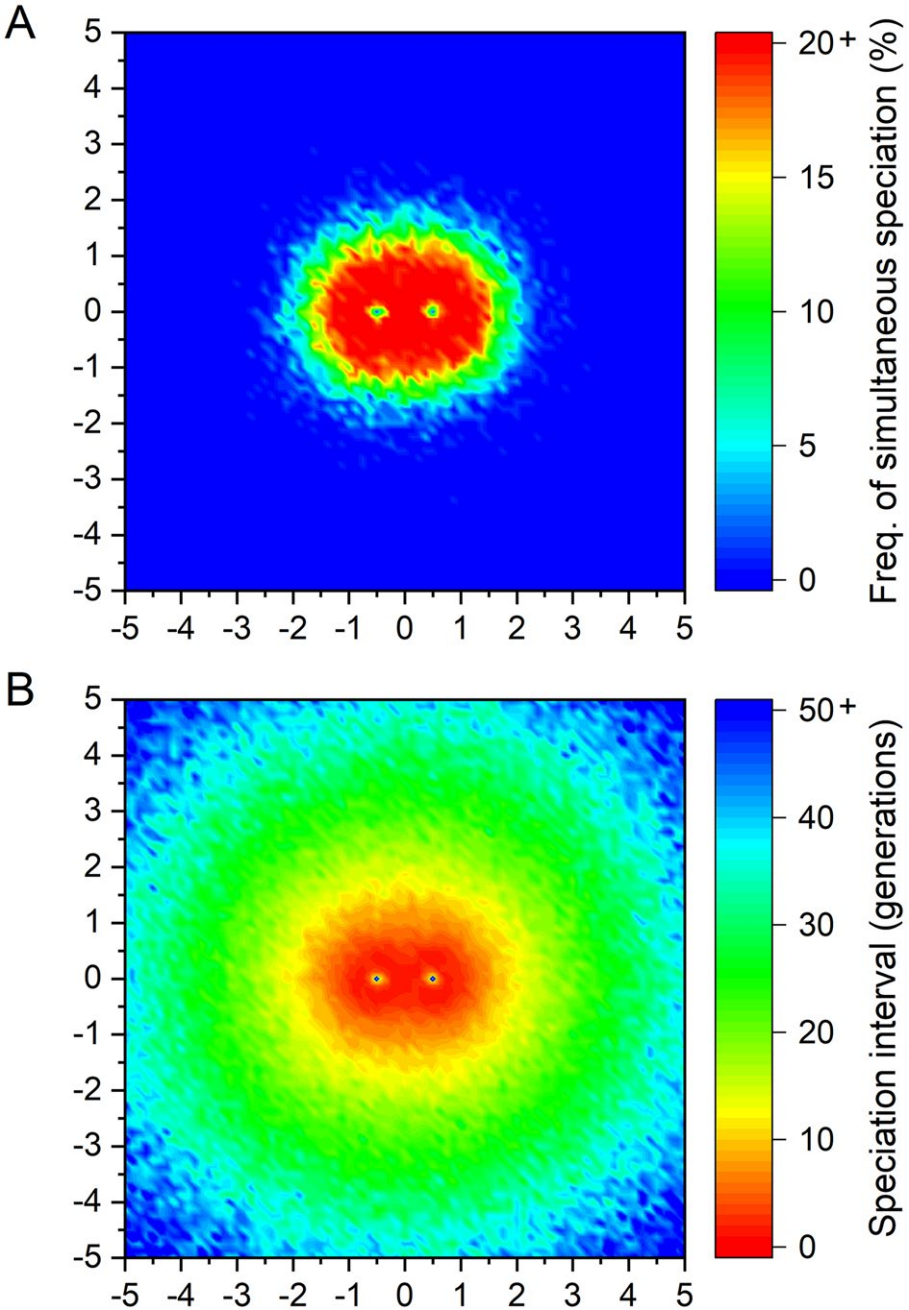
Effect of the geographical arrangement of islands on the frequency of simultaneous speciation (**A**) and the median speciation interval (**B**). Islands B and C are located at (− 0.5, 0) and (0.5, 0), respectively. The colour of each point is based on 20 simulations with island A placed at that point.

**Figure 3:**
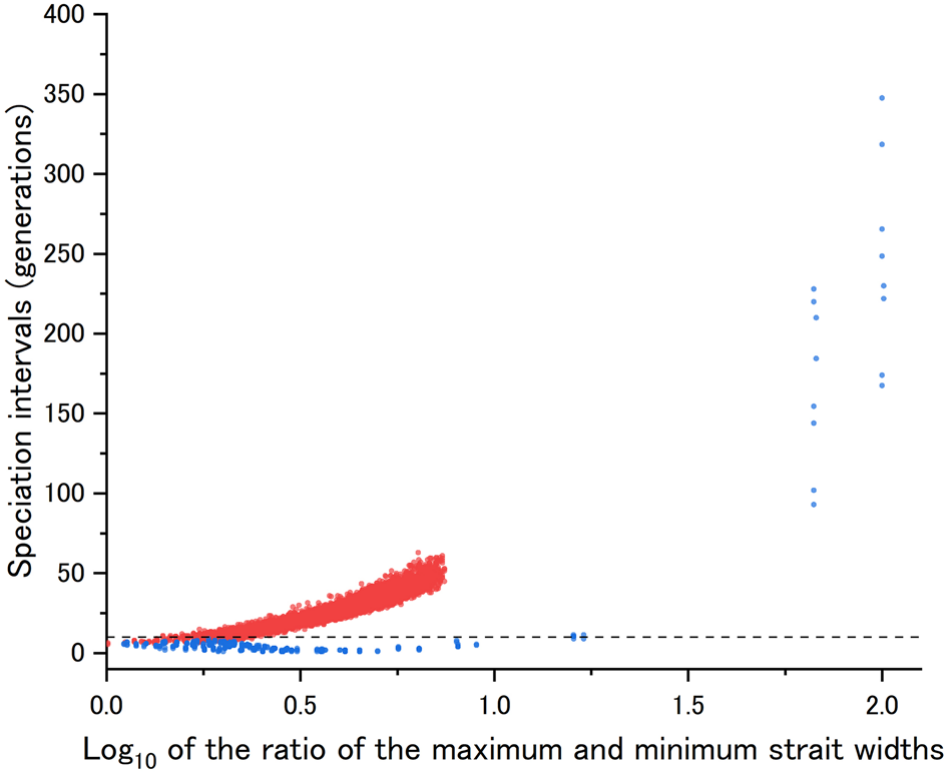
Effect of the presence of geographically isolated islands on the median speciation interval. The horizontal axis gives the log10 of longest strait width/shortest strait width, which is an indicator of the degree to which archipelagos contain geographically isolated islands. Red dots represent cases where island A is isolated, blue dots otherwise. The dashed line represents 10 generations, the criterion for simultaneous speciation. Each point is based on 20 simulations.

Figures 1, 2 and Fig. 3, Table 1, and Supplementary Table 1 suggest that there are regions in the parameter space that lead to frequent occurrences of simultaneous speciation. First, mutation rate (Fig. 1A) had little effect on speciation interval and frequency of simultaneous speciation. Second, the effects of population size also exhibited a similar trend in the absence of significant imbalances within the archipelago (Fig. 1B and Supplementary Table 1A). Although the presence of an extremely small population compared with others resulted in longer speciation intervals and a reduced frequency of simultaneous speciation, such speciation still occurred at a considerable frequency under these conditions (Supplementary Table 1B). Third, the strength of divergent natural selection within the archipelago (Fig. 1C) also did not substantially influence the results. Simultaneous speciation occurred frequently, regardless of whether local adaptation led to a reduction in dispersal ability or whether the stochastic loss of dispersal ability occurred. However, in cases of extremely strong divergent natural selection, the speciation intervals were significantly extended, and the frequency of simultaneous speciation was reduced. Furthermore, Table 1 reveals that an increase in the number of loci controlling dispersal ability extended the speciation interval with a negative impact on the frequency of simultaneous speciation. However, even with q = 15 loci, simultaneous speciation occurred at a non-negligible frequency. Lastly, the geographical arrangement of islands (Figs. 2 and 3) was found to have the most pronounced effect on the frequency of simultaneous speciation.

**Table 1:** Effect of the number of loci controlling dispersal ability (q) on the frequency of simultaneous speciation and speciation intervals.

MOdels	Freq. of simultaneous speciation	Speciation intervals		
		Min	Median	Max
q = 1	100%	0	2	5
q = 5	42%	0	12	64
q = 10	9%	0	73.5	3083
q = 15	8%	0	390	7726

*Each row is based on 100 simulations run with that setting. See Supplementary Note 1 for discussion.

When island A was located far enough away from islands B and C (i.e. when island A was geographically isolated), the speciation intervals increased and the frequency of simultaneous speciation decreased (Fig. 2). When island A was moderately close to islands B and C (i.e. when there were no geographically isolated islands in the archipelago), the speciation intervals were shorter and the frequency of simultaneous speciation increased (Fig. 2). When island A was too close to only one of the remaining two islands (i.e. when either island B or island C was relatively isolated), the speciation intervals again increased and the frequency of simultaneous speciation decreased (Fig. 2). In summary, the frequency of simultaneous speciation tended to be higher in cases of ordinary archipelagos, which include no geographically isolated islands and thus raise no questions about considering them as a single archipelago (Fig. 3). The reduction in the frequency of simultaneous speciation in archipelagos containing a geographically isolated island was more abrupt when island A (i.e., the island with a unique environment) was isolated than when island B or C (i.e., either of the islands with identical environments) was isolated.

In the analysis of the geographical arrangement of islands, we utilized settings designed to minimize computation time and enable efficient exploration of the parameter space. Critics might argue that the mutation rate (μ = 10^−4^) is too high, the population sizes (N_A_ = 100, N_B_ = 100, N_C_ = 100) are too small, and the divergent selection (DS = 0.7) is too strong. However, as shown in Fig. 1, these parameters have little effect on the frequency of simultaneous speciation. In fact, even 20 simulations using a more realistic parameter set (x =  − 1, y = 0, μ = 10^−5^, N_A_ = 5000, N_B_ = 5000, N_C_ = 5000, DS = 0.05) showed that, although the frequency of simultaneous speciation was reduced, it remained a common occurrence (frequency of simultaneous speciation 57%, minimum speciation interval 1, median 9, maximum 84; compared with the default parameter set with frequency of 100%, minimum speciation interval 0, median 1, maximum 5).

## Discussion

Our 133,300 simulations found regions in the parameter space where radiation is highly accelerated and even simultaneous speciation frequently occurs, resulting in long-lasting incipient species (Figs. 1–3, Table 1, Supplementary Table 1). Interestingly, those parameter regions were characterized by properties commonly found in ordinary archipelagos, not unusual ones; that is, there are no isolated islands that are separated from the other islands by wide straits, so that they are regarded as constituents of a single archipelago, and there are slight or moderate environmental differences among the islands.

**Figure 4:**
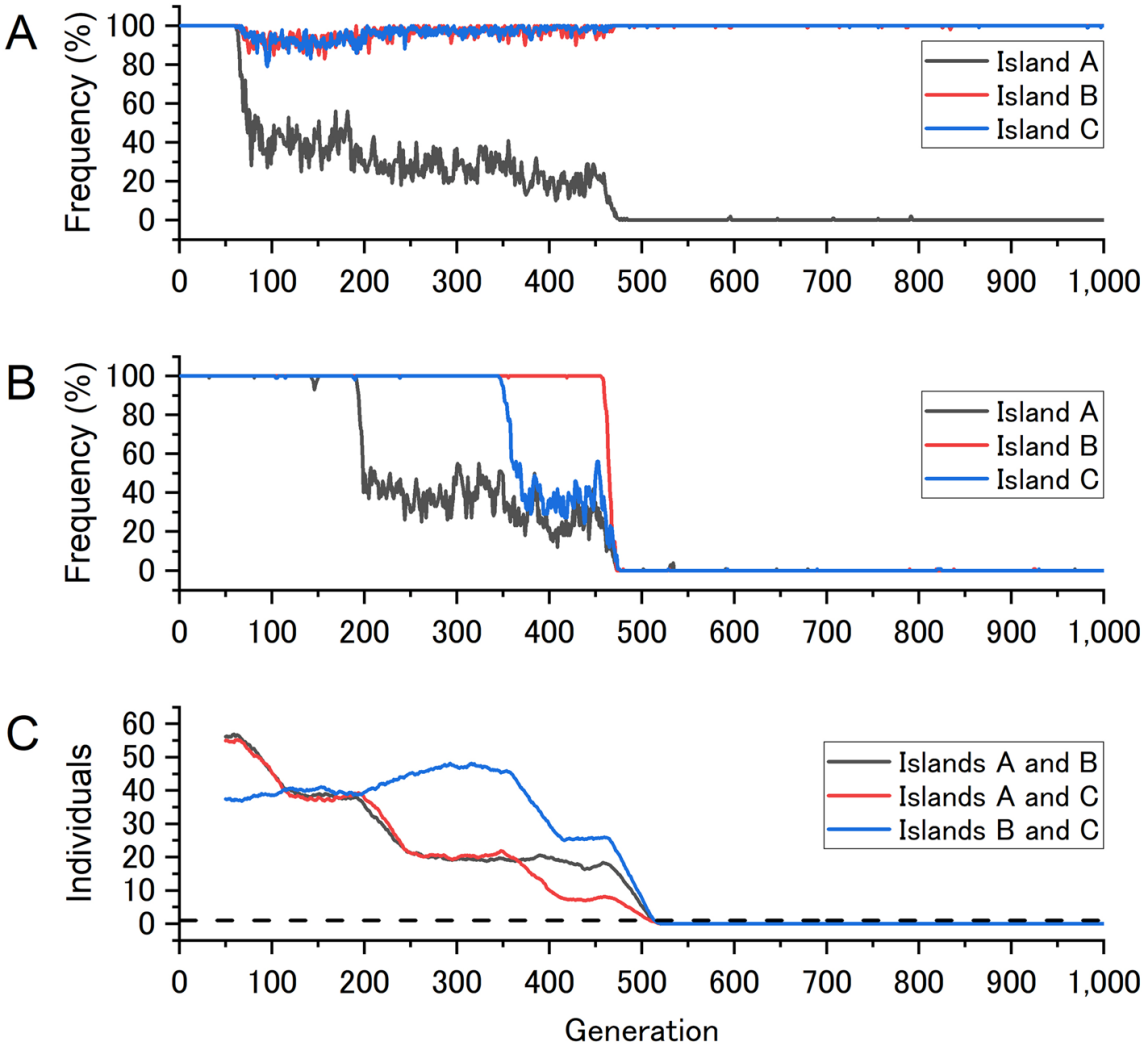
Evolution of local adaptations (**A**), reduction of dispersal ability (**B**) and occurrence of simultaneous speciation (**C**) in a representative simulation. The y-axes in panels A, B and C represent the frequency of primitive alleles adapted to low-altitude islands (islands B and C), the frequency of primitive alleles promoting dispersal and the average number of migrants moving between islands, respectively. The dashed line in panel C represents one migrant on average (OMPG).

To understand why simultaneous speciation occurs so frequently in this region of the parameter space, we conducted a detailed analysis of the simulation in a representative case (Fig. 4). The simulation shows that, by the 100th generation, there was considerable variation in allele frequencies among the islands at the locus associated with local adaptation, indicating that local adaptation was established (Fig. 4A). As mentioned earlier, once individuals were adapted to their birthplaces through local adaptation, selection favoured reduced dispersal. Indeed, around the 200th generation, the frequency of the dispersal-promoting allele began to decline on island A (black curve in Fig. 4B). Interestingly, however, this decline was rapid only at first, soon reaching a plateau. At this stage, the dispersal-promoting alleles were not completely lost from island A because of the continuous influx of these alleles from the other two islands. Next, around the 350th generation, mutation also occurred on island C, causing a rapid decrease in the frequency of dispersal-promoting alleles, but again a plateau was reached in a short time (blue curve in Fig. 4B). Then, the situation changed dramatically when mutation occurred on island B (around the 460th generation). On island B, the dispersal-promoting alleles rapidly declined and almost disappeared (red curve in Fig. 4B), while at the same time, on islands A and C, the frequency of dispersal-promoting alleles rapidly dropped from the plateau level towards zero, due to the loss of influx of migrants. As a result, the average numbers of individuals migrating between islands fell below 1 almost simultaneously for all island pairs, with a speciation interval of only five generations, indicating that simultaneous speciation occurred (Fig. 4C).

In summary, on ordinary archipelagos, which do not include extremely geographically isolated islands, no island population can attain a level of genetic isolation necessary for speciation to occur as long as other island populations continue to send out large numbers of migrants. Thus, complete isolation between any pair of islands must wait until the dispersal ability of the population on the final island with this ability begins to be lost; consequently, the timing of the onset of the allopatric speciation processes is inevitably highly synchronized within the archipelago. This mechanism operates similarly even when dispersal ability is lost stochastically. Although our simulations focused on the case with three islands, this synchronization mechanism is clearly applicable to archipelagos of four or more islands. We actually conducted simulations with ten islands and confirmed this point (see Supplementary Notes for details, including additional theoretical considerations such as the incorporation of continuously changing environments).

Our study discovered a key fact for understanding rapid adaptive radiation: as long as there is at least one island population that alleles reducing dispersal ability have not yet invaded, gene flow from such a population keeps individuals with high dispersal ability in all island populations within the archipelago. Once alleles reducing dispersal ability start to increase in such a population, all island populations simultaneously lose their dispersal ability and become isolated at almost the same time. Keeping this synchronisation mechanism in mind helps us understand why the frequency of simultaneous speciation decreases in certain cases. For example, in archipelagos with a geographically isolated island (see Figs. 2 and 3), the lower frequency of simultaneous speciation is due to the reduced likelihood of individuals with high dispersal ability being distributed across the entire archipelago in the later stages of the process. This perspective also helps explain the behaviours of models with unbalanced population sizes, quantitative genetic models and diploid models (see Supplementary Note 1).

Empirical observations on adaptive radiation strongly support our findings. First, most of the volcanic hotspot archipelagos where well-known cases of adaptive radiation are found are treated as a single archipelago, as they do not include islands that are extremely geographically isolated, and the environments are moderately different among the islands^34–37^. Second, most of the organisms on these archipelagos should fulfil the assumption of our simulations that dispersal ability is evolvable. This is because a large body of empirical evidence showing that dispersal ability in organisms is generally an evolvable trait has accumulated in recent years (for example, Refs.^29–33^). Among the cichlids of ancient lakes in Africa, the most diverse group inhabits rocky habitats scattered like islands on sandy shores. They seem to have evolved a behavioural tendency to remain in the same rocky habitat^48^. Finally, and importantly, the step involving the mechanism of adaptive radiation proposed here, whereby physically trivial barriers are augmented by reduced dispersal through natural selection, also has strong empirical support: in adaptive radiation on remote oceanic archipelagos, weak physical barriers, such as narrow straits or narrow lava fields, that would not function effectively for closely related species on a continent, often serve as boundaries of species distribution^34,35^. Similarly, for rock-dwelling cichlids in African ancient lakes, an instance where as little as 35 m of unsuitable habitat separates populations has been reported^48^. One of the things that most surprised Darwin during his stay on the Galapagos Islands was the difference in biota between islands that were so close to each other that they were visible to the naked eye^1^. Reduced dispersal ability on islands is sometimes counted as one of the ‘island syndromes’ (i.e. evolutionary trends common to island-dwelling organisms), but the mechanisms by which this occurs are not yet fully understood^29,31,33,49^. In the cases of island adaptive radiation, the mechanisms discussed here can make an important contribution to the reduction of dispersal ability and the consequent biological augmentation of physically trivial barriers.

As for the paradox of rapid radiation, it seems surprising that no-one had previously recognized the straightforward resolution based on the evolvable dispersal ability. This may be attributable to preconceptions arising from the classical biogeographical classification of speciation, which divides speciation into three categories—allopatric, parapatric and sympatric—based on the intensity of the physical barriers involved. Although recently a new classification scheme of speciation, ecological or non-ecological, has become more popular^4,30^, the biogeographical classification has been considered one of the most basic speciation classifications for the past 80 years or more (for example, Refs.^16,20^). Thus, most biologists have firmly believed that virtually all speciation can be classified into one of these three categories. However, one should note that, if the intensity of a physical barrier, such as a strait or mountain range, changes dynamically during the speciation process, then the speciation process can never be classified into one of these three categories. For example, we have regarded the speciation mechanism treated in this study as allopatric for convenience, given that the straits within the archipelago act as complete physical barriers late in the process. However, if one focuses on the initial stage of the process in which the straits do not or hardly impede gene flow, our mechanism can also be regarded as sympatric or parapatric speciation.

The geographical classification of speciation has been criticized for arbitrarily subdividing a continuum^50,51^, but more problematic is that this classification implicitly assumes invariant physical barrier strength. Consideration of whether the intensity of physical barriers remains static during the speciation process or changes dynamically due to the evolution of dispersal ability of organisms allows a more fundamental classification of speciation. There is then no reason to believe that the former classical ‘physical speciation’ is more common than the latter ‘bio-physical speciation’. Rather, recently accumulated empirical data (for example, Refs.^29–33^) support the opposite view. If we can discard long-held preconceptions about physical barriers, we can understand adaptive radiation much more straightforwardly than we do now.

## Methods

### Islands and island populations

The model used in this study consisted of three islands (or island-like habitats) and three island populations that could evolve into incipient species. We focused on radiation of three species because this is the simplest model for studying radiation.

Without loss of generality, we placed islands A, B and C at coordinates (x, y), (− 0.5, 0) and (0.5, 0), respectively, in a two-dimensional plane. As described below, the behaviour of our model is determined by the relative distance between islands, not the absolute distance between them. Therefore, it is sufficient to move only the coordinates of island A to examine the impact of the geographical arrangement of the islands on the results.

As mentioned above, we assumed that there are environmental differences among the three islands. We performed simulations assuming that there are two or three environment types in the archipelago, but both conditions produced essentially the same results (see Supplementary Table 2 and “Results” section); therefore, below we only show the results for cases with two environment types, which are more interesting in relation to nonadaptive radiation^18^. We assumed that islands B and C were in the same environment and that island A was in a different environment than that of B and C. For ease of understanding, in the following, islands B and C are provisionally described as low-altitude islands and island A as a high-elevation island. We also define the population sizes of islands A, B and C as N_A_, N_B_ and N_C_, respectively.

We assumed that the straits within the archipelago were considerably narrower than the distance from the nearest continent to the archipelago and hence did not act as effective barriers to the ancestral species, which supposedly had a strong capacity to disperse at the time of colonization. We therefore assumed that the ancestral species spread throughout the archipelago immediately after colonization. In addition, given that the colonization of remote archipelagos is likely to be carried out by very few founder individuals, we assumed that the ancestral species initially had no genetic variation, so that all loci were fixed to the primitive state (allele 0) in all island populations. Additionally, relaxing this assumption only shortens the time required for speciation to occur, without changing the conclusions (see Supplementary Table 3).

### Traits and their genetic bases

We performed simulations assuming haploid or diploid individuals, but the results were essentially the same (see Supplementary Table 2 and “Results” section). Therefore, only the results of the simpler haploid simulations are presented below.

We introduced two genetic traits: an adaptation-related trait and dispersal ability. We are not particularly interested in the process that leads to the evolution of local adaptation under gene flow, but rather in the subsequent evolution of dispersal ability. For this reason, we used the simplest model (i.e. the single-gene control model) for the traits related to adaptation, and the more realistic quantitative gene control model only for dispersal ability.

For the traits related to adaptation, we assumed that they are controlled by a single biallelic locus. For ease of understanding, here we assumed that the primitive state (allele 0) is adaptive on the low-altitude islands B and C, and the derived state (allele 1) is adaptive on the high-altitude island A, just as assumed in the taxon cycle theory^42^. However, the reverse setting (i.e. 0 is adaptive on A and 1 is adaptive on B and C) does not cause any essential difference to the results (see Supplementary Table 2 and “Results” section). Meanwhile, we assumed that dispersal ability was controlled by q biallelic loci. Individuals with all loci fixed to the primitive state (allele 0) were assumed to migrate to another island with a probability of M = 0.5 just before mating. Each additional derived allele (allele 1) was assumed to decrease the dispersal probability by M/q, and if all q loci were fixed to the derived state (allele 1), the individual was assumed to always remain on the island of its birth.

As the archipelago on which adaptive radiation takes place is usually small, we assumed that islands A, B and C were very close to each other and thus dispersal between them was not costly. However, when leaving the island of one’s birth, the choice of destination was assumed to depend on the geographical distances between the islands, with the closer island being more likely to be chosen. For example, the probability that an individual on island A would choose island B as its migratory destination was given by p_AB_ = (1/AB)/(1/AB + 1/AC) = AC/(AB + AC), where AB (AC) represents the geographical distance between islands A and B (C), and their reciprocals, 1/AB and 1/AC, are corresponding indicators of proximity between islands. The probabilities for the other island combinations were defined in similar ways. Although it is theoretically possible that an island becomes empty after migration, the probability of such chance extinction is negligibly small and we observed no such cases in our simulations.

### Mating

Mating occurs immediately after migration. To produce one offspring, two individuals from each island were drawn with replacement as parents and then mated. When choosing parents, the relative probability of being chosen is reduced by DS (0 < DS < 1) for locally maladapted individuals, so that the relative probabilities of being chosen are 1 and 1 − DS for locally adapted and maladapted individuals, respectively. In other words, DS is a measure of the strength of divergent natural selection within an archipelago.

For each locus, offspring randomly inherited the allele of either parent. Then, the allele was assumed to change to the other allele at a mutation rate µ.

We repeated the above process as many times as the number of offspring produced on each island. We set the number of offspring on each island equal to the initial population size (i.e. N_A_, N_B_, N_C_). Generations did not overlap, and all adults died at the end of each generation. Gene frequencies for the offspring generation were calculated at the birth of the offspring generation.

In summary, each generation began with the calculation of the gene frequencies of the island populations, followed by migration, and then mating occurred. Natural selection acted at the time of mating. All individuals of a generation died after mating and were replaced by the offspring generation. We ran simulations using this model under various parameter settings.

### Measurement of speciation intervals

The most straightforward way to determine whether simultaneous speciation occurs is to infer a phylogenetic tree using genome-scale molecular data generated by simulations. However, this involves a very large computational cost, making it virtually impossible to explore the parameter space. We therefore introduced a new measure, the speciation interval.

We suppose that three species arise from a single ancestral species through two dichotomous speciation events and define the speciation interval as the number of generations that have elapsed between the first and second speciation events. Measuring speciation intervals is difficult in the real world, but easy in simulation studies. We assume that a zero or very small speciation interval indicates the occurrence of simultaneous speciation. This is reasonable because, in such a case, there is not sufficient time for phylogenetic signals to accumulate in the inner branches of the phylogenetic tree, so that only multi-branched phylogenetic relationships can be reconstructed even using the largest amount of data^46^ (i.e. hard polytomy is observed).

To calculate the speciation interval, a criterion for identifying when speciation events began is also needed. We used the OMPG (one migrant per generation) rule^43–45^ as the criterion here. This rule states that population divergence by random genetic drift proceeds when the number of migrants falls below one per local population per generation. Keeping this rule in mind, we recorded the number of individuals that participated in mating after migrating from island A to island B or vice versa in each generation of the simulations. We divided this value by two, the number of populations involved, to convert it to a per-population mean. We continued to monitor the 50-generation moving average of this value throughout each simulation run. We did the same for the two other island pairs (A-C and B-C pairs). The resulting three moving averages were expected to decrease almost monotonically, due to selective pressure being exerted against dispersal after the establishment of local adaptation. Thus, the generation in which two of these three moving averages fell below one for the first time could be regarded as the first generation in which an island population reached sufficient genetic isolation, that is, the generation in which the first allopatric speciation event began. Similarly, the generation in which all three averages fell below one could be regarded as the first generation in which all island populations reached sufficient genetic isolation, that is, the generation in which the second allopatric speciation event began. We recorded the difference between these two generations as the speciation interval. Of course, our simulations do not meet many of the assumptions of the OMPG rule^45^, so the number of migrants required to stop population divergence might not be exactly one. However, for the purpose of evaluating the synchronisation of speciation timing, especially when dispersal ability changes over a short period, the exact estimate is not necessary, and using the OMPG rule would be sufficient. Furthermore, we judged that simultaneous speciation occurred when the speciation interval, albeit arbitrarily, was 10 generations or less. Given that within 100 generations is sometimes used as a criterion for contemporary evolution^47^, this is a very conservative criterion.

The method described here might be seen as problematic in that it considers the establishment of not intrinsic reproductive isolation but external geographical isolation as the time when the allopatric speciation event occurred. However, it should be noted that divergence times reconstructed using phylogenetic data generally refer to the latter, not the former. Thus, the use of this method is appropriate in the context of explaining the multi-branching phylogenetic patterns. General methods for estimating the timing of the establishment of intrinsic reproductive isolation have not been established, nor are they likely to be established in the future.

### Simulations

First, we systematically changed the mutation rate µ, population sizes N_A_, N_B_, and N_C_, and the intensity of divergent natural selection DS to assess their impact on the results, with 20 simulations run for each parameter setting. We used values arbitrarily chosen from the ranges of 10^−7^ to 10^−3^ for μ, 50 to 10,000 for N_A_, N_B_, and N_C,_ and 0.01 to 0.95 for DS. The chosen range for μ covers the estimated spontaneous mutation rates per locus (10^−6^ to 10^−4^) from empirical studies^52^. For parameters other than those systematically changed, we used the default values, x = 0, y = 0, μ = 10^−4^, N_A_ = 100, N_B_ = 100, N_C_ = 100, q = 1, DS = 0.7.

We then ran 100 simulations for each of q = 1, 5, 10 and 15 to assess the effect of the number of loci controlling dispersal ability. For the other parameters, we used default values.

Next, we assessed the effect of the geographical arrangement of the islands. The default value (x = y = 0), where island A is located at the midpoint between islands B and C, was only adopted as a starting point for the parameter space search because of its mathematical simplicity. In contrast, a geographical arrangement where island A is located to the left of − 0.5 or to the right of 0.5 on the x-axis and not too far away from the other two islands, for example, is more realistic. This is because many archipelagos well known for the occurrence of adaptive radiation, such as the Hawaiian, Galapagos and Macaronesian islands, are formed by volcanic hotspots, where islands tend to be arranged in a straight line due to the constant location of the volcanic hotspot and plate movement, with the elevation decreasing with the distance from the volcanic hotspot^34–37^. We systematically changed x and y (the coordinates of island A) and ran 20 simulations for each of 6,583 different geographical arrangements, including the above-mentioned realistic cases with the islands arranged in a straight line. In doing so, we used default values for parameters other than x and y. As an indicator of the degree to which archipelagos contain geographically isolated islands, we also used log10 of longest strait width/shortest strait width.

Finally, we conducted 20 simulations using a more realistic set of values than the default ones (x =  − 1, y = 0, μ = 10^−5^, N_A_ = 5000, N_B_ = 5000, N_C_ = 5000, q = 1, DS = 0.05).

We ran all simulations using Proc IML in SAS 9.4 (SAS, Cary, NC, USA), R 4.3.3 (R Core Team, Vienna, Austria), and the cutPhylo function from the R package RRPhylo^53^. We show the SAS and R codes we used as Supplementary Code 1 in the Supplementary Information. We drew all figures using OriginPro 2021b and 2023b (OriginLab, Northampton, MA, USA).

### Supplementary Information

Supplementary Information 1.

Supplementary Information 2.

## Data Availability

All data generated during this study are provided in a Supplementary Data File. The SAS and R codes used for the simulations are provided in the Supplementary Information.
