# Supplementary material for: Evolving dispersal ability causes rapid adaptive radiation
Source: Sci Rep. 2024 Jul 8;14:15734. doi: 10.1038/s41598-024-66435-w (PMC11231149; doi:10.1038/s41598-024-66435-w)
Supplement: Supplementary file 2 — Supplementary Information 2. [file 41598_2024_66435_MOESM2_ESM.pdf]

Evolving dispersal ability causes rapid adaptive radiation

Takeshi Yamasaki[1*] and Yutaka Kobayashi[2]

[1]Yamashina Institute for Ornithology, 115 Konoyama, Abiko, Chiba 270-1145, Japan ORCiD: https://orcid.org/0000-0002-9627-529X

[2]School of Economics and Management, Kochi University of Technology, 2-22 Eikokuji-cho, Kochi 780-8515, Japan

[*]Corresponding author: Takeshi Yamasaki (yamasaki@yamashina.or.jp)

Supplementary Information

Supplementary References

54 Del-Rio, G. *et al.* Birds of the Juruá River: extensive várzea forest as a barrier to terra firme birds. *J. Ornithol.* **162**, 565-577, https://doi.org/10.1007/s10336-020-01850-0 (2021).

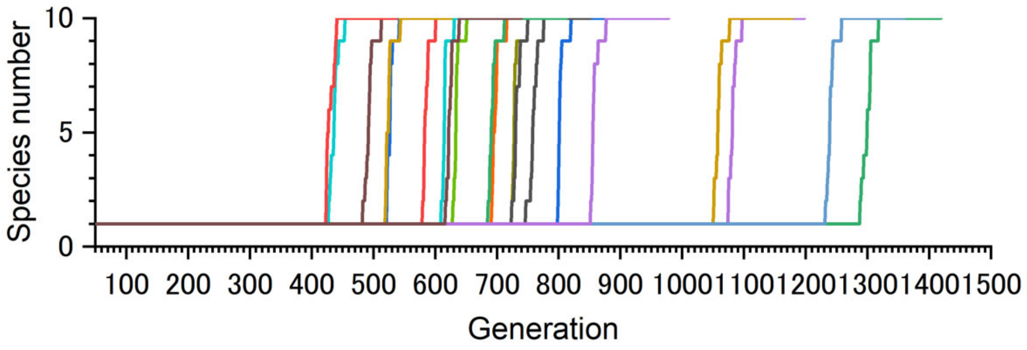

Supplementary Figure 1 | Dynamics of species numbers in 20 simulations using an extended model with 10 islands and 5 environments. Each line represents the result of one simulation. See Supplementary Note 3 and Supplementary Code 1 for details of the simulations and discussion.

Supplementary Table 1 | Effect of uneven population sizes on the frequency of simultaneous speciation and the speciation intervals. Each row is based on 20 simulations run using the setting given in the first column. See Supplementary Note 1 for discussion.

		Speciation intervals		
Population size	Freq. of simultaneous speciation	Min	Median	Max
A. 10 times difference				
N_A=100, N_B=1000, N_C=1000	100%	0	2	8
N_A=1000, N_B=100, N_C=1000	95%	0	2	12
B. 100 times difference				
N_A=100, N_B=10000, N_C=10000	30%	1	39	182
N_A=10000, N_B=100, N_C=10000	85%	0	2.5	69

Supplementary Table 2 | Frequency of simultaneous speciation and speciation intervals in more complex models. Each row is based on 20 simulations run using the model described in the first column. See Supplementary Code 1 for details of the simulations.

		Speciation intervals		
Models	Freq. of simultaneous speciation	Min	Median	Max
Three kinds of environments	100%	0	1.5	10
The ancestral type adapted to island A	100%	0	2	4
Diploid				
adaptation-related trait: primitive allele dominance				
dispersal ability: primitive allele dominance	100%	0	1	4
dispersal ability: codominance	90%	1	2.5	13
dispersal ability: derived allele dominance	95%	0	4	15
adaptation-related trait: codominance				
dispersal ability: primitive allele dominance	100%	0	1	3
dispersal ability: codominance	100%	0	2	4
dispersal ability: derived allele dominance	95%	0	4	12
adaptation-related trait: derived allele dominance				
dispersal ability: primitive allele dominance	100%	0	1	4
dispersal ability: codominance	95%	0	3	19
dispersal ability: derived allele dominance	95%	0	5	13

Supplementary Table 3 | Impact of genetic variation in the first generation on the time to the first speciation event, the frequency of simultaneous speciation and the speciation intervals. Each row is based on 20 simulations run using the setting given in the first column. See Supplementary Code 1 for details of the simulations.

	Time to the first speciation event				Speciation intervals		
Freq. of derived alleles	Min	Median	Max	Freq. of simultaneous speciation	Min	Median	Max
5%	107	321	3222	100%	0	2	4
10%	98	171.5	2856	100%	0	1	6
15%	89	128	1606	100%	0	2	4
20%	73	91	1403	100%	0	2	7

Supplementary Note 1 | Additional Discussion on Parameter Settings

As shown in Supplementary Table 1, significant imbalances in population sizes lead to a considerable decrease in the frequency of simultaneous speciation, although this is still not a rarity. This decline was especially severe when the population size on island A was extremely small. Because small populations have fewer opportunities for mutations, it is likely that, in this case, the population on island A will continue to release migrants until the later stages of the process. However, there are only a few migrants from the small population on island A, and they must migrate to islands with different environments, where they tend to fail to establish themselves. Consequently, the synchronization mechanism caused by the continuous influx of migrants is likely to become less effective. In contrast, when the population on island B or C is small, at least some of the migrants present in the later stages of the process can migrate to the other island with the suitable environment, thereby lessening the inhibition of the synchronization mechanism. Additionally, it should be noted that uneven population sizes not only affect the frequency of simultaneous speciation but also potentially influence the phenotypic diversification and the evolution of reproductive isolation mechanisms after the emergence of the three allopatric incipient species: evolution by random drift occurs more rapidly in small populations, while adaptive evolution accelerates in larger populations.

Table 1 also reveals that an increase in the number of loci controlling dispersal ability negatively impacts the occurrence of simultaneous speciation. The rather high number of loci ($q=15$), while still resulting in a non-negligible frequency of simultaneous speciation (8%), considerably increases the median interval between speciation events (390 generations). It should be noted that, when q is large, mutations must occur at all of the many loci to produce individuals with complete loss of dispersal ability. These mutations can occur in any island population, and the effect of reducing the dispersal ability of each mutated gene is very small. Therefore, in the later stages of the process, any island population should have a significant number of loci containing mutant genes that either arose in that island population or were introduced from other populations owing to the small effects of each mutant allele on reducing dispersal ability. In our model, simultaneous speciation occurs because an island population continues to release individuals with high dispersal ability in the later stages of the process, but such an island population is less likely to persist to these stages if q is large.

Finally, Supplementary Table 2 shows that, in the diploid simulations, a higher level of dominance of alleles reducing dispersal ability was associated with longer speciation intervals and a lower frequency of simultaneous speciation. This likely occurs because the effects of alleles reducing dispersal ability become more apparent with higher levels of dominance, making it less likely for individuals with high dispersal ability to persist in the later stages of the process.

Supplementary Note 2 | Non-adaptive Radiation

The theory developed here also provides the remarkable insight that a large number of types of environments are not necessary to trigger adaptive radiation: only two types of environments were sufficient to induce radiation of the three species in our simulations. These radiations included two ecologically identical species and one species that was ecologically different from them. This finding casts new light on the study of non-adaptive radiation. The term 'non-adaptive radiation' has been used for radiations involving many ecologically similar, allopatric species. Rundell and Price[18] inferred that patterns of non-adaptive radiation arose in a manner driven mainly by geographic isolation and genetic drift, rather than natural selection, and argued that non-adaptive radiation could secondarily shift to adaptive radiation through the acquisition of ecological differences and sympatry. However, our simulations show that it is not possible to make such inferences about the driving force from the pattern of non-adaptive radiation. This is because ecologically quite similar allopatric species can also arise quite commonly in radiations driven by natural selection, as in the populations on islands B and C in our simulations.

Supplementary Note 3 | Model Extensions

The model developed here can be extended in two directions. The first direction is to represent the environment as a continuous gradient rather than discrete islands. For example, African ancient lakes contain not only discrete habitats that can be considered as 'islands', such as rocky shores interspersed with coastal sand, but also environments that should be described as continuous gradients, for variables such as water transparency. Investigating what happens to ancestral species distributed in 2D space with continuously changing environments when dispersal ability is assumed to be evolvable is an interesting future research objective. It is conceivable that local adaptation might occur even under gene flow, potentially leading to an evolutionary reduction in the dispersal range of individuals. Gene flow between neighbouring populations would normally prevent speciation, but if weak physical barriers exist in 2D space, they could be strengthened, potentially leading to the emergence of incipient species. Indeed, a turnover phenomenon has been observed in Amazonian birds, where species are replaced by closely related ones across narrow rivers that would not normally serve as barriers, although this does not necessarily imply that speciation has occurred there[54].

The second direction for extending the model is to increase both the number of islands and the number of environments to four or more each. Actual examples of adaptive radiation typically occur under such complex conditions. Intuitively, it would seem that the synchronisation mechanism would continue to work effectively in such cases; however, it is crucial to validate this expectation by extending the model. Nevertheless, there are two major obstacles to this task.

The first obstacle concerns the probabilities of selecting migration destinations. The original model defined these probabilities based on an inter-island proximity index, namely, the inverse of geographical distance. However, when a large number of islands are placed in a given area, it is inevitable that some island pairs will end up being very close to each other. For these pairs of islands, the proximity index becomes so high that it is virtually impossible for the ancestral species living on them to migrate to other islands. This contradicts the assumption that ancestral species with high dispersal ability can easily move anywhere within the small archipelago. To overcome this problem, it would be effective to generalise the selection probabilities of migration destinations, making them independent of the inter-island proximity index.

The other obstacle is the complexity of determining the number of species. Because the original model involved only three islands, this could be easily accomplished by monitoring the three average numbers of migrants: one can conclude that the first speciation occurred when two averages met the OMPG criterion, and the second when all three averages met the OMPG criterion. However, as the number of islands increases, applying such a simple determination method becomes challenging. This is because the number of island combinations increases explosively and various species with diverse distributions emerge until the final state of one species per island is reached. A

single-linkage (friends-of-friends) cluster analysis can be used to solve this problem. By conducting this analysis using the average numbers of migrants as similarities, clusters linked by more than one migrant can be correctly identified as a single species.

We implemented the second direction of extension, involving many islands and environments, and developed code to handle a case with 10 islands and 5 environments (see Supplementary Code 1). Running 20 simulations with equal probabilities for selecting destination islands, we consistently observed an explosive increase in the number of species from one to ten within a short period of approximately 20 to 30 generations (see Supplementary Fig. 1).

Here, we define the speciation interval as the number of generations between one speciation event and the next. If the speciation interval is 10 generations or fewer, we consider simultaneous speciation to occur. With this definition, we found that the frequency of simultaneous speciation was 90%, with a minimum speciation interval of 0 generations, a median of 2 and a maximum of 18. In all simulations, the speciation intervals were short and the frequencies of simultaneous speciation were high until the ninth species was produced (frequency of simultaneous speciation 100%, minimum speciation interval 0, median 1, maximum 8). However, for unknown reasons, after the ninth species emerged, it took longer for the tenth species to arise (frequency of simultaneous speciation 20%, minimum speciation interval 2, median 13, maximum 18). The pattern up to the ninth species closely resembled the results of the three-island model (frequency of simultaneous speciation 100%, minimum speciation interval 0, median 1, maximum 5), although comparisons of these results may not be entirely appropriate due to different settings of probabilities for choosing destination islands. Understanding the detailed behaviour of the extended model is an important task for future research.

Supplementary Note 4 | Japanese Version of the Manuscript

急速な適応放散は分散力の進化によって起きる

山崎剛史 [1*] 小林 豊 [2]

[1] 山階鳥類研究所 〒270-1145 千葉県我孫子市高野山 115 ORCiD: https://orcid.org/0000-0002-9627-529X

[2] 高知工科大学経済・マネジメント学群 〒780-8515 高知県高知市永国寺町 2-22

[*] 責任著者 山崎剛史 (yamasaki@yamashina.or.jp)

要旨

ダーウィン以来の長い研究史にもかかわらず、急速な適応放散の根底にあるメカニズムはまだ十分に理解されていない。これまでに構築された理論はいずれも特殊な仮定を必要とし、幅広い分類群に見られる実際の事例を包括的に説明できるものはない。ここで私たちは、この問題に対するシンプルな理論的解決を提案する。それは、分散力の進化可能性という、経験的によく支持される仮定を加えることで、古典的な適応放散の群島モデルをより現実的なモデルに拡張することである。進化可能な分散力を導入した個体ベースのシミュレーションにより、島（または島状のハビタット）間の環境の不均質性によって分散力の進化的減少がもたらされることが示された。しかし、適応放散が報告されている実際の群島でよく見られるように、島がほぼ均等に分布している場合には、いくつかの島集団で始まった分散力の減少は、他島からの継続的な移民の流入によってすぐに停止した。これらの島集団における分散力減少のプロセスは、最も長いあいだ、高い分散力を維持し、移民の放出を続けていた最後の島において、分散力の減少が始まったとき、ほぼ同時に再開された。その後、分散力はすべての島集団で急速に失われた。つまり、多数の異所性発端種の同時進化の頻発は、私たちのシミュレーションにおいて、通常の群島の特性の必然的帰結であった。本研究は、一見複雑に見える急速な放散のプロセスが、分散力の進化的減少という単純なメカニズムによって駆動されている可能性を強く示唆している。

適応放散とは、利用されていない資源すなわち生態学的機会[6,9]へのアクセスをきっかけに、単一の祖先種から生態学的に著しく多様な子孫種が進化することである[1–8]。適応放散は短期間に多くの種を生み出すことが多いが、このような急速な種分化を可能にするメカニズムはまだよく分かっていない[10]。最近のゲノムスケールの系統解析[11–14]は、この問題をさらに複雑にしてしまった：これらの解析は、急速な放散の最も極端なかたちである多分岐の系統学的パターンをさまざまな分類群において見出したのである。これらの例では、連続する種分化イベント間の間隔があまりに短すぎて統計的にゼロと区別できない。このようなポリトミーはすでに多くの遺伝子座を用いて研究されているため、データを追加しても解像度が向上することはないだろう。このような性質を持つポリトミーは「ハードポリトミー」と呼ばれ、データ不足による低解像度を意味する「ソフトポリトミー」とは区別される[15]。ハードポリトミーは、これまでに鳥類[11]、クジラ[13]、ショウガ[12]、マメ[14]などで発見されている。また、マラウイ湖のシクリッド、タンガニーカ湖のシクリッドにもハードポリトミーの可能性のあるノードが見つかっている[11]。

適応放散の経験的研究を行う研究者の多くは、群島モデル[2,5,7]とよばれる理論を受け入れ、物理的バリアによって遺伝子流動が妨げられることで起きる異所的種分化が適応放散における種分化の主要な様式であるとみなしてきた（例えば、文献 2,5,16–18）。しかし、この理論のもとで急速な放散と、その最も極端なかたちである同時種分化を説明することは難しい。なぜなら、異所的種分化による適応放散は、多数の物理的バリアが同時またはほぼ同時に出現するか、あるいは多数の物理的バリアを越えた急速かつ一過性の種の分布拡大を必要とするが[15,18]、これらのアドホックなシナリオはどちらも生物学的に非現実的に思えるからだ。

これに対し、適応放散の理論的研究を行う研究者は、もっぱら非異所的な種分化モデルに基づき、急速な放散と同時種分化の説明を試みてきた（例えば、文献 10,19–22）。しかし、そうした努力にもかかわらず、急速な放散の説明は、依然として困難を伴ったままである。非異所的な種分化モデルでは、種分化の原動力として、しばしば分岐自然選択が主

要な役割を果たす。この前提の下では、一般に、放散における最初の種分化は、生態学的に最も大きく異なる 2 つの娘種を生み出すと期待される。そして、これら 2 つの娘種のそれぞれが 2 つの孫娘種へと分岐する際には、それらの孫娘種間の生態学的な違いは必然的に娘種間の違いより小さくならざるを得ない。同様にして、分岐選択の強度は種分化が繰り返し起きるにつれて弱まっていき、その結果、次の種分化イベントまでの待ち時間はむしろ長くなっていくはずなのである。この問題は最近 Martin と Richards[10] によって「急速な放散のパラドックス」と名付けられた。

理論的には、非異所的種分化モデルの下でも、急速な放散や同時種分化が起こり得ることが知られているものの、これには特殊な条件の充足が必要である。例えば、Gavrilets らの理論[19] は、未知の原因による、突然かつ急激な分散力の減少を必要とするだけでなく、パラメータセッティングへの極端な依存性もあり、適応放散は容易には起きない[20]。Gavrilets と Vose の理論[21] は、食植性昆虫の同所的種分化に適用される特殊な種分化メカニズム[23] に依存している。Bolnick の理論[22] は、適応ダイナミクス理論[24] に基づいており、著者自身も認めているように、パラメータセッティングが非現実的である。Martin と Richards[10] は、トランスポーター仮説、シグナル複雑性仮説、適応景観連結性仮説、多様性が多様性を生む仮説、柔軟な幹／可塑性第一仮説のもとで、急速な放散が起きる可能性について議論した。しかし、これらの理論の前提についての経験的証拠は、たとえあるにしても、ごくわずかな分類群でしか得られていない。このため、これらの個別理論の組み合わせによって、植物[12,14]、軟体動物[25]、昆虫[26]、魚類[27,28]、爬虫類[27]、鳥類[11,27]、哺乳類[13,27] など、多岐にわたる分類群で観察されている急速な放散の実例をどの程度説明できるのかは、依然として不明のままである。

ここで私たちはこのジレンマを解決するシンプルなアイデアを提案する。つまり、私たちは、経験的研究を行う研究者が繰り返し採用してきた古典的な適応放散の群島モデルに、分散力の進化可能性を組み込んだ。物理的バリアの強度は、海峡の幅や山脈の高さなどの物理的特性だけでなく、生物の分散力にも依存していることに注目してほしい。鳥や

昆虫の翼、果実のトゲ、浮力をもたらす果実の構造など、分散力に大きな影響を与える特徴の多くは遺伝的基盤を持つ。複数の物理的バリアが同時多発的に出現したり、物理的特性を同時多発的に変化させたりすることは考えにくくても、祖先種の地域集団が同時多発的に分散力を失う進化を起こすことはあり得るだろう。つまり、ほとんどの生物群で満たされるであろう分散力の進化可能性の仮定（例えば、文献 29–33 を見よ）には、多数の物理的バリアの同時またはほぼ同時の成立による急速な放散のシナリオの生物学的妥当性を著しく改善する効果が期待されるのである。

改変されたシナリオの概要は次の通りだ。まず、大陸から遠く離れた小群島に侵入できた祖先種は、長距離の飛行を行う能力、長時間の漂流に耐える能力など、初期値では高い分散力を持っていたに違いない。次に、適応放散の引き金となる生態的機会[6,9]は、多くの種類の未利用資源の存在を意味するが、そのような資源の相対的な頻度は、通常、適応放散の舞台となる群島のサブエリア間で異なるだろう。例えば、大規模な適応放散が起こったことでよく知られるハワイ諸島、ガラパゴス諸島、マカロネシア諸島は、いずれもかなりの降水量のある高海抜の島と乾燥した低海抜の島の両方で構成されている[34–37]。このような環境の不均質性から生じる分岐自然選択は、たとえ祖先種の高い分散力に起因する強い遺伝子流動があったとしても、局所適応の進化を引き起こす可能性がある[38]。そして、いったん局所適応が確立されれば、個体には自分の生まれたサブエリアに適応する傾向が生じる。このような条件下では、個体は分散によって適応していない環境に出会う機会が増えるため、結果として分散力に負の選択圧が働く。このため、もし分散力が進化可能な特性なら、自然選択によってそれは必然的に減少するはずである[38–41]。そのような分散力の減少の結果、以前には機能していなかった物理的バリアが、その物理的特性を変えないままに強化されて、最終的にサブエリア間の遺伝的隔離がもたらされる。そして、私たちのシミュレーションが以下で明らかにするように、島間の遺伝的隔離は、通常の条件下において高度に同期して起き、多くの発端種が同時多発的に出現するに至るのである。

私たちは、本研究で、分散力の進化可能性を仮定した修正群島モデルを用い、個体ベー

スのシミュレーションを実施した。その目的は、私たちが予想したように、(1)局所適応が進化し、(2)分散力が減少し、(3)高度に同期した物理的バリアの強化が起きる、すなわち同時種分化が起きる可能性がどのくらいあるのかを評価することである。

結果

ここで私たちは 3 つの島からなる群島を分析した。それぞれの島は局所的には環境的に均質だが、島間の環境は異なると仮定する。すべての島が同じ環境条件であるため、分岐選択が働かないという、単一の環境タイプしかない些細なケースは除外する。環境タイプを 2 つにするか 3 つにするかは、結果に本質的な影響を与えないことがわかったため(方法参照)、以下では、環境タイプが 2 つで島が 3 つある場合のみを扱う。私たちは同一環境にある 2 つの島を島 B、C、環境的にユニークな島を島 A とよぶ。理解を容易にするため、前二者を低海抜の島、後者を高海抜の島と仮に表現する。祖先種は高海抜または低海抜のどちらの条件に適応していてもよいが、この初期条件は結果に本質的な影響を及ぼさないことがわかった(方法参照)。そこで、以下では、タクソンサイクル理論[42]にならい、祖先種が低海抜の島(島 B、C)に適応していることを仮定する。タクソンサイクル理論は、島の生物相の特徴を説明するためのもので、島に新しく到達した祖先種は低海抜の沿岸地帯に適応する傾向を示すが、やがては高海抜地帯へと侵出することを主張する。

局所適応の進化に関する私たちの最初の予測は、分岐自然選択が非常に弱い場合を除き、さまざまなパラメータセッティングで実施したシミュレーション(合計 133,300 回)のほぼすべてにおいて満たされていた。高海抜の島と低海抜の島の集団は、初期の強い遺伝子流動にもかかわらず、ほとんどの場合、最終的にはそれぞれの環境に適応した別々の遺伝子の固定へと至った。ただし、分岐自然選択が非常に弱い場合にはそのような局所適応が進化しないこともあった。次に、分散力の進化的減少に関する私たちの 2 番目の予測は、すべてのシミュレーションにおいて常に満たされていた。分岐自然選択によって局所適応が成立する場合には、分散促進対立遺伝子の頻度はすべての島集団で低下し、最終的

には、どの島のペアについても、局所集団あたり世代あたりの平均移民数は必ず 1 より小さくなって、OMPG（One Migrant Per Generation）ルールに基づき（方法と文献 43–45 参照）、3 つの異所性発端種が生じたと判断された。一方、分岐自然選択が弱く、局所適応が成立しない場合についても、シミュレーションを長く続ければ、分散型遺伝子の機会的喪失により、最終的には必ず 3 つの異所性発端種が生じた。しかし、このプロセスによって生じた発端種は寿命が短く、機会的浮動によって分散力が再び増加する結果として融合し、1 種に戻る事例がよく観察された。

一方、同時種分化に関する 3 つ目の予測が満たされるかどうかは、パラメータの設定次第であった。なお、ここで私たちは、恣意的だが非常に保守的に、1 回目と 2 回目の種分化のあいだの間隔が 10 世代以下であったときに同時種分化が起きたと仮定した（方法と文献 46,47 参照）。私たちが探索したパラメータの範囲について言えば、上記の定義による同時種分化は 13,023 回（9.8%）起きており、これらの事例の中には、1 回目と 2 回目の種分化がまったく同一の世代に起きた例（種分化間隔=0）さえもが含まれていた（648 回のシミュレーション、全体の 0.5%）。

図 1〜3、表 1、補足表 1 は、パラメータ空間中に同時種分化の頻発をもたらす領域が存在することを示唆している。まず、突然変異率（図 1A）は、種分化間隔と同時種分化の頻度にほとんど影響を与えなかった。また、集団サイズの影響も群島内に著しい不均衡がない場合には同様の傾向を示した（図 1B、補足表 1A）。他と比べて極端に小さな集団があるときには、種分化間隔が伸び、同時種分化の頻度が低下したが、そのときでさえ、同時種分化はかなりの頻度で起きていた（補足表 1B）。次に、群島内の分岐自然選択の強さ（図 1C）も結果に大きな影響を与えなかった。局所適応の成立によって分散力が低下する場合でも、分散力の機械的喪失が起きる場合でも、同時種分化は高い頻度で起きていた。ただし、例外として、群島内に極端に強い分岐自然選択が働く場合については、種分化間隔がかなり伸び、同時種分化の頻度が低下した。さらに、表 1 は、分散力をコントロールする遺伝子座の数の増加が、種分化間隔を伸ばし、同時種分化の頻度にネガティブな影響を与

えることを明らかにした。ただし、q=15 のときでさえ、同時種分化は無視できない頻度で起きていた。最後に、島の地理的配置（図 2、3）は、同時種分化の頻度に最も顕著な影響を及ぼすことが明らかになった。

島 A が島 B、島 C から十分に離れた位置にある場合（つまり、島 A が地理的に孤立している場合）には、種分化間隔が伸び、同時種分化の頻度が低下した（図 2)。島 A が島 B、島 C に適度に近接する場合（つまり、群島内に地理的に孤立した島がない場合）には、種分化間隔が短くなり、同時種分化の頻度が増加した（図 2)。島 A が残り 2 島のうちのどちらか一方のみに近づきすぎた場合（つまり、島 B もしくは島 C が相対的に孤立している場合）には、再び種分化間隔が伸び、同時種分化の頻度が低下した（図 2)。まとめると、同時種分化の頻度は、群島内に地理的に孤立した島がなく、それらの島々を単一の群島とみなすことに何の疑問の生じない、ごくふつうの群島の場合には高くなる傾向がある（図 3)。群島内に地理的に孤立した島があるときの同時種分化頻度の減少は、島 A（すなわち、独自の環境を持つ島）が孤立しているときの方が、島 B または C（すなわち、同じ環境を持つ島のいずれか）が孤立しているときより、急速であった。

島の地理的配置の解析では、計算時間を最小化し、パラメータ空間を効率的に探索できるようにデザインした設定を用いた。突然変異率（$\mu=10^{-4}$）が高く、集団サイズ（N_A =100, N_B =100, N_C =100）が小さく、分岐自然選択が強すぎる（DS=0.7）との批判があるかもしれない。しかし、図 1 に示した通り、これらのパラメータは同時種分化の頻度にほとんど影響を与えない。実際、より現実的なパラメータセット（$x=-1$, $y=0$, $\mu=10^{-5}$, N_A=5000, N_B=5000, N_C=5000, DS=0.05）を用いた 20 回のシミュレーションでも、同時種分化は、頻度が低下したものの、依然としてごくふつうにみられる現象のままだった（同時種分化の頻度 57%、種分化間隔最小値 1、中央値 9、最大値 84；デフォルトパラメータセットでの同時種分化の頻度 100%、種分化間隔最小値 0、中央値 1、最大値 5)。

考察

私たちが実施した133,300回のシミュレーションは、パラメータ空間内に、放散が高度に加速され、同時種分化さえもが頻繁に起きる結果、長期的に存続する発端種が生み出される領域を発見した（図1〜3、表1、補足表1）。面白いことに、このパラメータ領域は、特殊な群島ではなく、ごくふつうの群島に一般的に見られる特性を表していた。つまり、他の島々と広い海峡で隔てられた孤立した島がないために、単一の群島とみなされ、島間の環境はわずかに、または、ほどよく異なっているという特性である。

パラメータ空間内のこの領域で、なぜ同時種分化が頻発するのかを理解するため、私たちは代表的なケースのシミュレーションを詳細に分析した（図4）。このシミュレーションでは、100世代目までに局所適応に関連する遺伝子座の対立遺伝子頻度にかなりの島間変異が生じ、局所的適応が成立したことが示された（図4A）。前述したように、局所適応によっていったん個体が生誕地に適応するようになると、分散を減少させる選択が働くようになった。実際、200世代目ごろ、島Aにおいて、分散促進対立遺伝子の頻度が低下し始めた（図4Bの黒い曲線）。しかし、興味深いことに、この減少が急速だったのは最初だけで、それはすぐに停滞期に突入した。この段階で島Aから分散促進対立遺伝子が完全に失われなかったのは、他の2島からこれらの対立遺伝子が継続的に流入していたせいである。次に、350世代目ごろ、島Cでも突然変異が起き、分散促進対立遺伝子の頻度が急速に減少したが、再び短期間で停滞期に突入した（図4Bの青い曲線）。その後、島Bで突然変異が起こると状況は一変した（460世代目頃）。島Bでは、分散促進対立遺伝子が急速に減少してほぼ消失し（図4Bの赤い曲線）、同時に島Aと島Cでも、移民の流入がなくなったため、分散促進対立遺伝子の頻度が停滞期のレベルからゼロに向かって一挙に減少した。結果として、島間を移動する平均個体数は、すべての島ペアについてほぼ同時に1を下回り、種分化間隔はわずか5世代になり、同時種分化が起きたことが示された（図4C）。

まとめると、地理的に極端に孤立した島を含まない、ごくふつうの群島では、他の島集団が大量の移民を送り出している限り、どの島集団も種分化に必要なレベルの遺伝的隔離

にまで到達できないのである。このため、どの島ペアについても完全な隔離が起きるには、分散力を最後まで維持し続けた島集団でこの能力が減少し始めるのを待たなくてはならないのだ。結果として、異所的種分化プロセスが始まるタイミングは必然的に群島内で高度に同期する。このメカニズムは分散力が機会的に失われる場合でも同じように働く。今回のシミュレーションは 3 つの島のケースを対象としたが、この同期のメカニズムが 4 つ以上の島からなる群島にも適用可能であることは明らかだ。私たちは実際に島の数を 10 島に増やしたシミュレーションを実施し、この点を確認した（詳細については補足説明参照。そこでは、連続的に変化する環境の組み込みなど、追加の理論的考察を行った）。

私たちの研究は、急速な適応放散を理解するための鍵を発見した：それは、分散力を低下させる対立遺伝子が未侵入のままの島集団が 1 つでも残っている限り、そこからの遺伝子流動により、群島内のすべての島集団に分散力の高い個体が存在し続けるという事実だ。そのような島集団で分散力を低下させる対立遺伝子がいったん増え始めると、すべての島集団がほぼ同時に分散力を失い、ほぼ同時に隔離される結果になる。このメカニズムが種分化の同期の原因であることを念頭に置けば、特定の事例で同時種分化の頻度が下がる理由も容易に理解できるようになるだろう。例えば、地理的に孤立した島を含む群島で同時種分化の頻度が下がるのは（図 2、3 参照）、プロセスの終盤において、分散力の高い個体が群島全体に分布している状況が生じにくくなるせいである。不均衡な集団サイズのモデル、量的遺伝子モデル、二倍体モデルの挙動についても、この観点からの説明が可能である（補足説明 1 参照）。

適応放散についての経験的観察は、今回の発見を強く支持している。まず、適応放散のよく知られた事例が見られる火山性ホットスポット群島の多くは、地理的に極端に孤立した島を含まないために単一の群島として扱われており、島間の環境が適度に異なっている[34–37]。次に、これらの群島に生息する生物のほとんどは、分散力が進化可能であるという、私たちのシミュレーションの前提を満たしているはずである。生物の分散力が一般に進化可能な特徴であることを示す経験的証拠が近年数多く蓄積されているからだ（例え

ば、文献 29–33）。アフリカの古代湖のシクリッドについては、砂浜に島状に点在する岩場に生息するグループが最も多様化しているが、彼らは同じ岩場にとどまり続ける行動学的傾向を進化させているらしい[48]。最後に、重要なこととして、些細な物理的バリアが自然選択による分散の減少によって強化されるという、ここで提案した適応放散のメカニズムに含まれるステップにもまた、強い経験的裏付けがある：海洋性の離島における適応放散では、狭い海峡や狭い溶岩原など、大陸の近縁種では有効に機能しないような弱い物理的バリアが、しばしば種の分布境界として機能しているのである[34,35]。アフリカの古代湖の岩礁性シクリッドについても、わずか 35 メートルの生息不適地が個体群を隔てている例がある[48]。ガラパゴス諸島に滞在していたダーウィンが最も驚いたことの一つは、肉眼で見えるほど近接している島々の間で生物相に違いがあることであった[1]。島における分散力の減少は、島に住む生物に共通してみられる進化的傾向である「島症候群」の一つに数えられることもあるが、それが起きるメカニズムはまだ十分に理解されていない[29,31,33,49]。島の適応放散の事例では、ここで論じたメカニズムが、分散力の減少と、その結果として起きる些細な物理的バリアの生物学的強化に対して、重要な貢献をなしている可能性がある。

　急速な放散のパラドックスについて言えば、進化可能な分散力という、ストレートな解決策にこれまで誰も気付かなかったことは驚くべきことだろう。これには、種分化の古典的な生物地理学的分類から生じた先入観が影響していたのかもしれない。この分類は、関与する物理的バリアの強度に基づき、種分化を異所的、側所的、同所的種分化の 3 カテゴリーに分ける。最近では、生態学的か非生態学的かという、新たな種分化の分類図式がポピュラーになってきたが[4,30]、生物地理学的分類は、過去 80 年以上にもわたり、最も基本的な種分化の分類の一つだとされてきた（例えば、文献 16,20）。このため、生物学者の多くは、事実上、すべての種分化は、これら 3 つのカテゴリーのいずれかに分類できると固く信じ込んできた。しかし、もし、種分化プロセスの最中に海峡や山脈などの物理的バリアの強度が動的に変化したなら、その種分化プロセスをこれら 3 つのカテゴリーの 1 つに

分類することは決してできないことに注目すべきである。例えば、私たちは、本研究で扱った種分化のメカニズムを、プロセスの後期に群島内の海峡が完全な物理的バリアとして機能することを考慮して、便宜上、異所的種分化とみなしたが、もし、このプロセスの初期段階に注目するなら、海峡が遺伝子流動を妨げないか、ほとんど妨げないため、このメカニズムを同所的または側所的種分化とみなすこともできるのである。

種分化の生物地理学的分類は連続体を恣意的に細分化しているとの批判を受けてきたが[50,51]、それよりずっと深刻な問題はこの分類が物理的バリアの強度の不変性を暗黙のうちに仮定していることだ。種分化プロセスの最中に物理的バリアの強度が静的なままなのか、生物の分散力の進化によって動的に変化するのかを考慮することは、より根本的な種分化の分類を可能にする。そして、前者の古典的な「物理的種分化」が、後者の「生物物理的種分化」よりも一般的だと信じるべき理由は何もない。むしろ最近の経験データの蓄積はその逆の見方を支持している（例えば、文献 29–33）。もし私たちが物理的バリアに関する長年の先入観を捨て去ることができれば、私たちはいまよりずっと簡単に適応放散を理解することができるだろう。

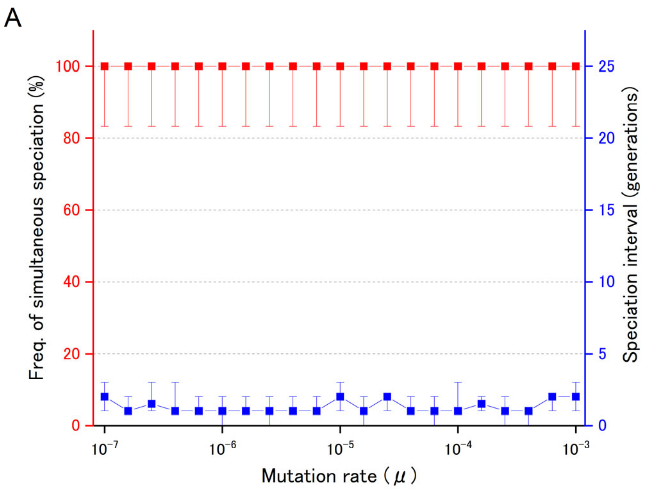

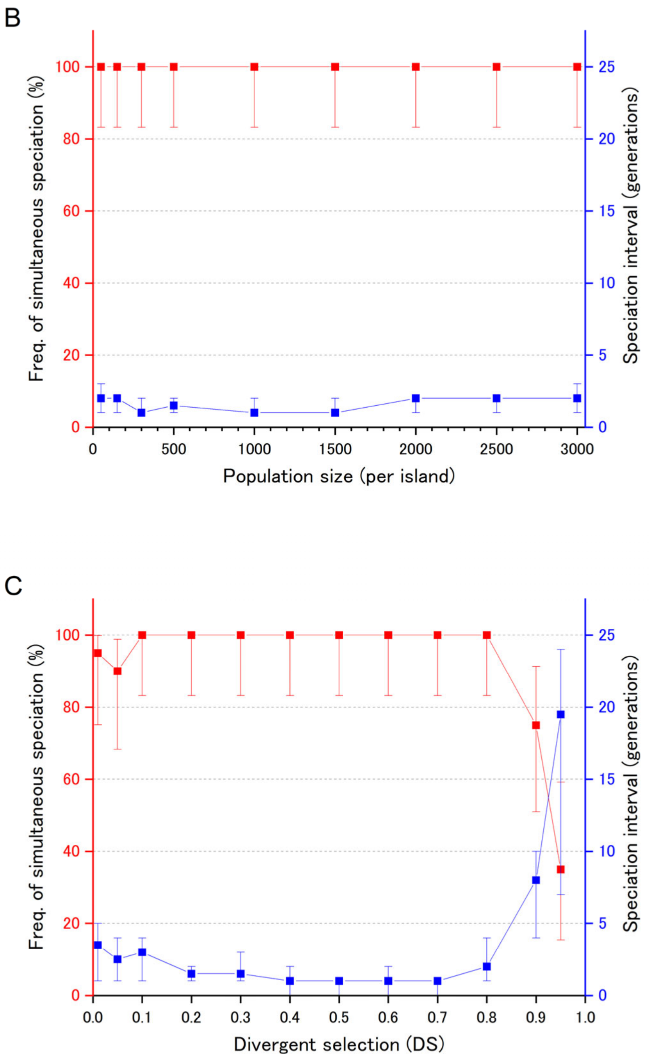

図 1｜突然変異率 μ（A）、集団サイズ（B）、分岐自然選択の強さ DS（C）が同時種分化頻度（赤）と種分化間隔中央値（青）に及ぼす影響。各点は、その設定で実行した 20 回のシミュレーションに基づく。エラーバーは 95%信頼区間を表す。

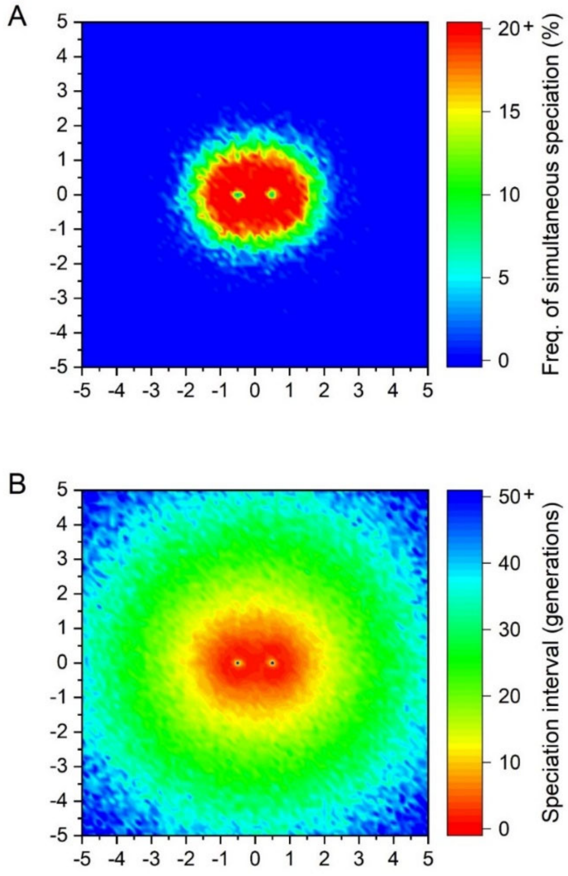

図 2｜島の地理的配置が同時種分化頻度（A）と種分化間隔中央値（B）に及ぼす影響。

島 B と島 C はそれぞれ (-0.5, 0) と (0.5, 0) にある。各点の色は、島 A をその点に置いたときの 20 回のシミュレーションに基づく。

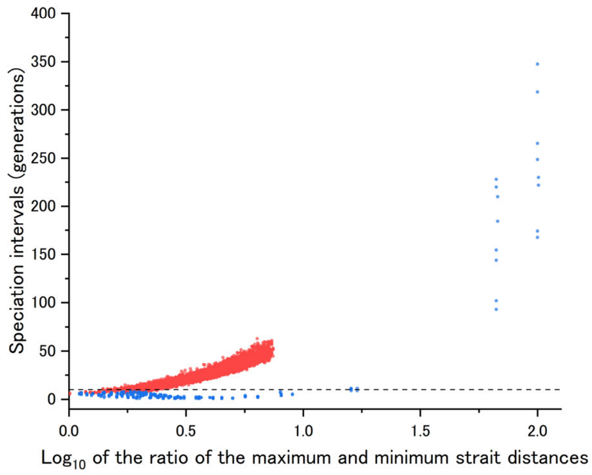

図 3｜地理的に孤立した島の存在が種分化間隔中央値に及ぼす影響。横軸は、最長海峡幅/最短海峡幅の常用対数を表し、群島が地理的に孤立した島を含む度合いを示す指標である。赤点は島 A が孤立している場合、青点はそれ以外の場合を表す。破線は同時種分化の基準である 10 世代を表す。各点は 20 回のシミュレーションに基づく。

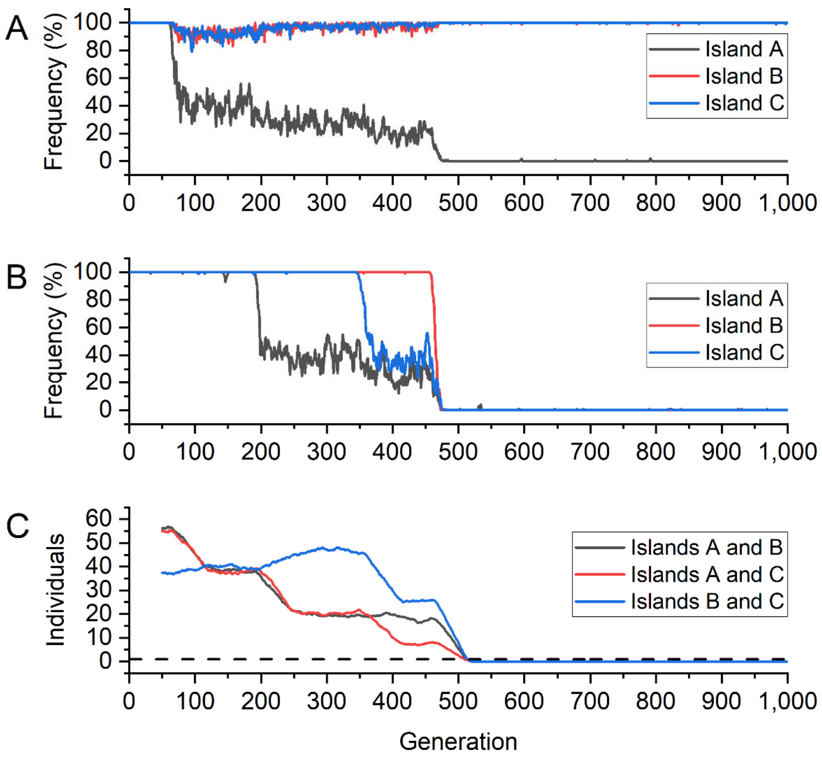

図 4｜代表的なシミュレーションにおける局所適応の進化（A)、分散力の減少（B)、同時種分化の発生（C)。パネル A、B、C の y 軸は、それぞれ、低海抜の島（島 B、C）に適応した原始的対立遺伝子の頻度、分散を促進する原始的対立遺伝子の頻度、島間を移動した個体の数の平均を表す。パネル C の破線は平均 1 個体（OMPG）を表す。

表 1｜分散力を支配する遺伝子座の数（q）が同時種分化頻度と種分化間隔に及ぼす影響。各行は、その設定で実行した 100 回のシミュレーションに基づく。考察については補足説明 1 参照。

		Speciation intervals		
Models	eous speciation	Min	Median	Max
q=1	100%	0	2	5
q=5	42%	0	12	64
q=10	9%	0	73.5	3083
q=15	8%	0	390	7726

方法

島と島集団

本研究で用いたモデルは、3 つの島（または島状のハビタット）と、発端種へと進化する可能性のある 3 つの島集団からなる。3 種の放散に注目したのは、それが放散を研究する上で最も単純なモデルだからだ。

私たちは、一般性を失うことなく、島 A、B、C を 2 次元平面上の座標(x, y)、(-0.5, 0)、(0.5, 0)に配置した。後述するように、本モデルの挙動は、島間の絶対距離ではなく、相対距離に依存して決まっている。したがって、地理的配置が結果に及ぼす影響を調べるには、島 A の座標のみを動かすだけで十分である。

前述の通り、私たちは、3 つの島のあいだに環境の違いがあることを仮定した。私たちは群島内に 2 種類または 3 種類の環境があると仮定してシミュレーションを実施したが、どちらの条件からも本質的に同じ結果が得られたため（補足表 2 と結果参照)、以下では非適応放散 [18] との関係でより興味深い、環境の種類数が 2 つの場合の結果のみを示した。私たちは、島 B と島 C は同一の環境にあるが、島 A は島 B、C とは異なる環境にあると仮定した。以下では、理解を容易にするため、島 B、C を低海抜の島、島 A を高海抜の島と仮に表現する。私たちはまた、島 A、B、C の集団サイズをそれぞれ N_A , N_B , N_C と定義する。

私たちは、群島内の海峡が、最も近い大陸から群島までの距離よりもはるかに狭く、入植の時点で高い分散力を持っていたと想定される祖先種に対して、有効なバリアとして機

能していなかったと仮定した。このため、私たちは、祖先種が入植後すぐに群島の全域に広がったと仮定した。また、遠隔の群島への侵入はきわめて少数の創始者個体で行われる可能性が高いことから、祖先種は初期状態では遺伝的変異を持たず、どの島集団についても、すべての遺伝子座が原始的状態（対立遺伝子 0）に固定されていると仮定した。なお、この仮定を緩和しても種分化が起きるまでの時間が短くなるだけで、結論は変わらない（補足表 3）。

形質とその遺伝的基盤

私たちは一倍体または二倍体の個体を仮定してシミュレーションを実施したが、結果は本質的に同じだったので（補足表 2 と結果参照）、以下では、よりシンプルな一倍体のシミュレーションの結果のみを示した。

私たちは、適応に関係する形質と分散力という 2 つの遺伝形質を導入した。私たちは、遺伝子流動下で局所適応が生じるまでのプロセスにとくに興味があるわけではなく、むしろその後の分散力の進化の方により強い興味がある。このため、私たちは、適応に関係する形質については最も単純なモデル（すなわち、単一遺伝子制御モデル）を使用し、分散力についてのみ、より現実的な量的遺伝子制御モデルを使用した。

適応に関係する形質については、単一遺伝子座の 2 つの対立遺伝子によって制御されていると仮定した。理解を容易にするため、ここではタクソンサイクル理論[42]で想定されたのと同じく、原始状態（対立遺伝子 0）が低海抜の島 B、C に適合し、派生状態（対立遺伝子 1）が高海抜の島 A に適合するとした。しかし、逆の設定（すなわち、0 が島 A に適合し、1 が島 B、C に適合する設定）を用いても、結果に本質的な違いは生じない（補足表 2 と結果参照）。一方、分散力は、q 個の遺伝子座によって制御され、それぞれの座に 2 つの対立遺伝子があることを仮定した。すべての遺伝子座が原始状態（対立遺伝子 0）に固定されている個体については、交配の直前に M=0.5 の確率で他の島へと移動する。派生的な対立遺伝子（対立遺伝子 1）が 1 つ増えるごとに分散の確率は M/q ずつ減少し、すべ

ての遺伝子座が派生状態（対立遺伝子 1）に固定されると、その個体は生まれた島に必ず留まり続ける。

適応放散の舞台となる群島はふつう小さいため、島 A、B、C は互いに非常に近くにあり、島間の分散にはコストがかからないと仮定した。ただし、生誕地の島から離れる際、行先の選択は島間の地理的距離に依存して決まり、より近くの島の方が選ばれる可能性が高いと仮定した。例えば、島 A の個体が島 B を移住先に選ぶ確率は、p_{AB}=(1/AB)/(1/AB+1/AC)=AC/(AB+AC)で与えた。ここで AB（または AC）は島 A と島 B（または島 C）の地理的距離を表し、その逆数である 1/AB と 1/AC は、対応する島間の近さの指標である。私たちは、他の島の組み合わせについても、同様の方法で確率を定義した。理論的には、移住によって島が空になることがあり得るが、そのような偶然の絶滅の確率は無視できるほど小さく、今回のシミュレーションでそのようなケースは見られなかった。

交配

交配は移住の直後に起きる。1 個体の子を作るため、各島から 2 個体が重複を許して親として選ばれ、交配した。親を選ぶ際、局所的に不適合な個体については、選ばれる相対確率が DS（0<DS<1）だけ低下する。従って、選ばれる相対確率は局所適応個体が 1、局所不適応個体が 1–DS となる。つまり、DS は群島内の分岐自然選択の強さの指標である。

子は、各遺伝子座について、どちらか一方の親の対立遺伝子をランダムに受け継ぐ。その後、突然変異率 μ でこれらの対立遺伝子が他方の対立遺伝子へと変化すると仮定した。

私たちは以上のプロセスを各島で生まれる子の数だけ繰り返した。各島の子の数は、初期個体数（すなわち、N_A , N_B , N_C ）と等しく設定した。世代は重複しておらず、すべての成体が各世代の終わりに死亡する。子世代の遺伝子頻度は、子世代の誕生時に計算した。

まとめると、各世代は島集団の遺伝子頻度の計算から始まり、移住が続き、交配が起き

る。自然選択は交配時に作用する。その世代のすべての個体は交配後に死亡し、子世代によって置き換えられる。私たちは、さまざまなパラメータセッティングのもとでこのモデルを用い、シミュレーションを実行した。

種分化間隔の測定

同時種分化が起きたかどうかを判断するための最も素直な方法は、シミュレーションによって生成したゲノムスケールの分子データを使って系統樹を推定することである。しかし、これは非常に大きな計算コストを要求するため、パラメータ空間の探索が事実上不可能になってしまう。そこで、私たちは、種分化間隔という新しい尺度を考案した。

私たちは、2 回の二分岐的種分化イベントによって 1 つの祖先種から 3 つの種が生じたと仮定し、最初の種分化イベントと 2 回目の種分化イベントの間に経過した世代数を種分化間隔と定義する。種分化間隔の測定は、現実世界では困難だが、シミュレーション研究では容易である。私たちは、種分化間隔がゼロか非常に小さいことが、同時種分化が起きたことを示すと仮定する。これが合理的であるのは、このような場合、系統樹の内部の枝に系統学的シグナルが蓄積される時間が十分でないため、最大量のデータを用いても多分岐の系統関係しか再構築できなくなる（すなわちハードポリトミーが観察される）状況が生じるからである[46]。

種分化間隔を計算するためには、種分化イベントが起きた時期を特定するための基準がさらに必要になる。私たちは、ここで、その基準として、OMPG（one migrant per generation）ルール[43-45]を用いた。このルールは、移民の数が 1 局所集団あたり 1 世代あたり 1 個体を下回ると、機会的遺伝的浮動による集団分岐が進行するとするものである。このルールを念頭に、私たちは、シミュレーションの各世代において、島 A から島 B へ、またはその逆方向の移動を行ったうえで交配に参加した個体の数を記録した。私たちはその値を、関与する集団の数である 2 で割り、集団あたりの平均値へと変換した。私たちは各シミュレーションの実施中、この値の 50 世代移動平均をモニターし続けた。私たちは、

他の 2 組の島ペア（A-C ペアと B-C ペア）についても同様のことを行った。結果として得られた 3 つの移動平均は、局所適応の確立後、分散を不利にする自然選択の作用により、ほぼ単調に減少すると期待された。したがって、これら 3 つの移動平均のうち 2 つが初めて 1 を下回った世代は、ある 1 つの島集団が十分な遺伝的隔離に到達した最初の世代、すなわち、最初の異所的種分化イベントが起きた世代とみなすことができる。同様に、3 つの移動平均がすべて 1 を下回った世代は、すべての島集団が十分な遺伝的隔離に到達した最初の世代、つまり、2 回目の異所的種分化イベントが起きた世代とみなすことが可能である。私たちはこれら 2 つの世代間の差を求め、それを種分化間隔として記録した。もちろん、私たちのシミュレーションは、OMPG ルールの仮定 [45] の多くを満たしていないため、集団の分岐を止めるのに必要な移民の数は厳密には 1 個体ではないかもしれない。しかし、種分化のタイミングの同期を評価するという目的に照らせば、とくに分散力が短期間に変化する場合には、正確な推定は必要でなく、OMPG ルールを使用するだけで十分だろう。さらに、私たちは、恣意的ではあるものの、種分化間隔が 10 世代以下であったときには、同時種分化が起きたと結論した。現代進化の基準として 100 世代以内が用いられることがあることを考えれば [47]、これは非常に控えめな基準である。

ここで述べた方法については、内的な生殖隔離の確立時期ではなく、外的な地理的隔離の成立時期を異所的種分化イベントが起きた時期とみなしている点が問題視されるかもしれない。しかし、系統学的データによって復元される分岐年代は、一般に前者ではなく、後者であることに留意すべきである。従って、この方法の使用は多分岐の系統学的パターンを説明するという文脈に照らして適切である。内的な生殖隔離の成立時期を推定するための一般的な方法は確立されていないし、今後も確立されなさそうである。

シミュレーション

私たちはまず、突然変異率 μ、集団サイズ N_A、N_B、N_C、分岐自然選択の強さ DS が結果に及ぼす影響を評価するため、それらを体系的に変化させ、それぞれのパラメータセッテ

ィングごとに 20 回のシミュレーションを実施した。μ については 10^{-7}〜10^{-3} の範囲、N_A、N_B、N_C については 50~10000 の範囲、DS については 0.01〜0.95 の範囲から任意に選んだ値を使用した。使用した μ の範囲は、経験的研究から推定された遺伝子座あたりの自然突然変異率（10^{-6}〜10^{-4}）をカバーしている [52]。体系的に変化させたパラメータ以外については、デフォルト値である x=0, y=0, μ=10^{-4}, N_A=100, N_B=100, N_C=100, q=1, DS=0.7 を使用した。

私たちは次に、分散力を制御する遺伝子座の数の影響を評価するため、q=1, 5, 10, 15 のそれぞれに対し、100 回のシミュレーションを実施した。そのほかのパラメータについては、デフォルト値を使用した。

続いて私たちは、島の地理的配置の影響を評価した。島 A が島 B と島 C の中点に位置するというデフォルト値（x=y=0）は、数学的にシンプルであることから、パラメータ空間探索の出発点として採用されたにすぎない。これに対し、例えば、島 A が x 軸上の-0.5 より左側か、0.5 より右側に位置し、かつ、他の 2 島から離れすぎていない地理的配置はより現実的である。ハワイ諸島、ガラパゴス諸島、マカロネシア諸島のように、適応放散が起きたことでよく知られる群島の多くは、火山性ホットスポットによって形成されており、それらの群島では、火山性ホットスポットの位置の不変性とプレート運動の結果として、島が一直線に並び、火山性ホットスポットから離れるほど標高が低くなる傾向があるからである [34–37]。私たちは x と y（島 A の座標）を体系的に変化させ、島がまっすぐに並ぶ前述の現実的なケースを含む 6,583 通りの地理的配置のそれぞれに対し、20 回のシミュレーションを実施した。その際、x と y 以外のパラメータについては、デフォルト値を使用した。私たちはまた、群島が地理的に孤立した島を含む度合いを示す指標として、最長海峡幅／最短海峡幅の常用対数を用いた。

最後に私たちは、デフォルト値より現実的な値のセット（x=−1, y=0, μ=10^{-5}, N_A=5000, N_B=5000, N_C=5000, q=1, DS=0.05）を用いて 20 回のシミュレーションを実施した。

すべてのシミュレーションは、SAS 9.4 (SAS, Cary, NC, USA)の Proc IML、R 4.3.3 (R Core

Team, Vienna, Austria)、R パッケージ RRPhylo[53] の cutPhylo 関数を用いて実施した。使用したSAS と R のコードは補足コード 1 として補足情報に示した。すべての図は、OriginPro 2021b および 2023b (OriginLab, Northampton, MA, USA)によって描かれた。

データの入手可能性

本研究で生成したすべてのデータを補足データファイルとして提供する。シミュレーションに使用した SAS と R のコードは、補足情報に掲載する。

2023 年 9 月 14 日受付　2024 年 7 月 1 日受理

引用文献

…

謝辞

コメントをいただいた長谷川政美氏、丸山敦氏、布施静香氏、三上修氏、米澤隆弘氏、森本元氏、北村亘氏、鹿野雄一氏、鈴木大氏、二人の匿名の査読者に感謝する。本研究のきっかけとなった南部琉球とハワイ諸島の生物相の観察にご協力いただいた、松原始氏、高木昌興氏、梶田学氏、岡本卓氏、内山春雄氏、北條政利氏、Bishop Museum, Honolulu に感謝する。原稿の校閲を行った Edanz（https://jp.edanz.com/ac）に感謝する。計算機資源は京都大学学術情報メディアセンター（ACCMS）と九州大学情報基盤研究開発センター（RIIT）から提供された。本研究は、日本学術振興会科研費番号 23K23952、22H02689、18K06397 の支援を受けて実施した。

著者の貢献

T.Y.は研究の構想を練り、T.Y.と Y.K.はモデルの開発を行い、T.Y.はコードの記述とシミュレーションを行い、T.Y.と Y.K.は結果の解釈と原稿の執筆を行った。

利益相反

著者らは利益相反がないことを宣言する。

追加情報

…

補足情報

補足引用文献

…

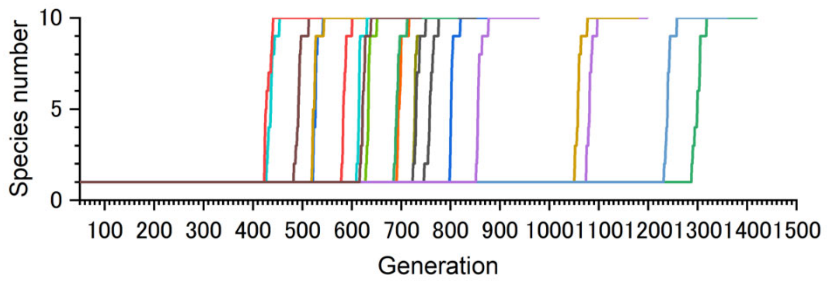

補足図 1｜10 島 5 環境の拡張モデルを用いた 20 回のシミュレーションにおける種数の変化。それぞれのラインは 1 回のシミュレーションの結果を表す。シミュレーションの詳細と考察については補足説明 3 と補足コード 1 参照。

補足表 1｜不均等な集団サイズが同時種分化頻度と種分化間隔に及ぼす影響。各行は、最初の列に示した設定で実行した 20 回のシミュレーションに基づく。考察については補足説明 1 参照。

Population size	Freq. of simultaneous speciation	Speciation intervals		
		Min	Median	Max
A. 10 times difference				
N_A=100, N_B=1000, N_C=1000	100%	0	2	8
N_A=1000, N_B=100, N_C=1000	95%	0	2	12
B. 100 times difference				
N_A=100, N_B=10000, N_C=10000	30%	1	39	182
N_A=10000, N_B=100, N_C=10000	85%	0	2.5	69

補足表 2｜より複雑なモデルにおける同時種分化頻度と種分化間隔。各行は、最初の列に示したモデルを用いて実行した 20 回のシミュレーションに基づく。シミュレーションの詳細については補足コード 1 参照。

Models	Freq. of simultaneous speciation	Speciation intervals		
		Min	Median	Max
Three kinds of environments	100%	0	1.5	10
The ancestral type adapted to island A	100%	0	2	4
Diploid				
adaptation-related trait: primitive allele dominance				
dispersal ability: primitive allele dominance	100%	0	1	4
dispersal ability: codominance	90%	1	2.5	13
dispersal ability: derived allele dominance	95%	0	4	15
adaptation-related trait: codominance				
dispersal ability: primitive allele dominance	100%	0	1	3
dispersal ability: codominance	100%	0	2	4
dispersal ability: derived allele dominance	95%	0	4	12
adaptation-related trait: derived allele dominance				
dispersal ability: primitive allele dominance	100%	0	1	4
dispersal ability: codominance	95%	0	3	19
dispersal ability: derived allele dominance	95%	0	5	13

補足表 3｜第一世代の遺伝的変異が最初の種分化が起きるまでの時間、同時種分化頻度、種分化間隔に及ぼす影響。各行は、最初の列に示した設定を用いて実行した 20 回のシミュレーションに基づく。シミュレーションの詳細については補足コード 1 参照。

Freq. of derived alleles	Time to the first speciation event			Freq. of simultaneous speciation	Speciation intervals		
	Min	Median	Max		Min	Median	Max
5%	107	321	3222	100%	0	2	4
10%	98	171.5	2856	100%	0	1	6
15%	89	128	1606	100%	0	2	4
20%	73	91	1403	100%	0	2	7

補足説明 1｜パラメータ設定に関する追加の議論

補足表 1 に示したように、集団サイズに著しい不均衡がある場合、同時種分化は依然としてまれな現象ではないものの、その頻度はかなり低下した。この低下は島 A の集団サイズが極端に小さいときにとくに激しかった。個体数の少ない集団は突然変異が生じる機会が少ないので、このケースでは、プロセスの終盤まで移住個体を排出し続けるのは島 A の集団であることが多いだろう。しかし、島 A の小さな集団からの移民は数が少なく、環境の違う島へ侵入しなければならないため、定着に失敗する傾向もある。結果として、移民の継続的流入による同期のメカニズムが働きにくくなるのだろう。これに対して、島 B や島 C の集団サイズが小さい場合、プロセスの終盤の移民の少なくとも一部は好適な環境の他島に侵入できるので、同期メカニズムの阻害の程度はより小さくなる。なお、不均等な集団サイズは、同時種分化の頻度だけでなく、3 つの異所性発端種が生じた後の表現型の分化や生殖隔離機構の進化にも影響を与える可能性があることに留意すべきだ。機会的浮動による進化は小集団で、適応進化は大集団で速く進む。

また、表 1 は、分散力をコントロールする遺伝子座の数の増加が同時種分化の発生にネガティブな影響を与えることを明らかにしている。遺伝子座数がかなり多い場合（q=15）でも同時種分化は無視できない頻度で起きてはいたものの（8%)、種分化間隔の中央値はかなり長くなっていた（390 世代)。q が大きい場合、分散力を完全に失った個体が出現するためには、多数の遺伝子座のすべてにおいて突然変異が起きる必要があることに注目すべきだ。これらの突然変異はどの島集団でも同様に起こり得るうえ、個々の突然変異遺伝子の分散力低下効果は非常に小さい。このため、プロセスの終盤には、どの島集団も、その島集団で生じたか、あるいは、分散力低下効果が低いために他集団から流入した突然変異遺伝子を含む遺伝子座が、かなりの数になっているはずである。私たちのモデルでは、プロセスの終盤に高い分散力を持つ個体を排出し続ける島集団があるために同時種分化が起きるが、q が大きいと、そのような島集団がその段階まで存続しにくくなるのだろう。

最後に、補足表 2 は、二倍体のシミュレーションについて、分散力を低下させる対立遺

伝子の優性の度合いが高いほど、種分化間隔が伸び、同時種分化の頻度が下がることを示した。そのような場合には、分散力を低下させる効果が顕在化しやすくなるため、プロセスの終盤に高い分散力を維持したままの個体が残存しにくくなる傾向が生じるのだろう。

補足説明 2｜非適応放散

ここで展開した理論は、適応放散を引き起こすには、必ずしも多くの種類の環境が必要なわけではないという注目すべき洞察も与えている。私たちのシミュレーションでは、3 種の放散を導くには、2 種類の環境だけで十分だったのである。これらの放散には、生態学的に同一の 2 種と、それらと生態学的に異なる 1 種が含まれていた。この発見は、非適応放散の研究に新たな光を投げかけている。「非適応放散」という用語は、生態学的に類似した多数の異所性の種からなる放散に対して使われてきた。Rundell と Price[18] は、非適応放散のパターンが、自然選択ではなく、主に地理的隔離と遺伝的浮動によって生じたと推測し、非適応放散は二次的に生態学的な違いと同所性を獲得することで適応放散に移行する可能性があると論じた。しかし、私たちのシミュレーションによれば、非適応放散のパターンから、駆動力についてこのような推測を行うことはできない。生態学的に酷似した異所性の種は、今回のシミュレーションの島 B、C の集団のように、自然選択によって駆動されている放散でも、ごくふつうに生じ得るからである。

補足説明 3｜モデルの拡張

ここで開発したモデルは、2 つの方向に拡張することができる。1 つ目の方向性は、環境を離散的な島ではなく、連続勾配として表現することだ。例えば、アフリカの古代湖は、沿岸部の砂地に点在する岩礁帯のように「島」とみなせる離散的なハビタットだけでなく、水の透明度など、連続勾配として表現すべき環境も含んでいる。分散力の進化可能性を仮定したとき、環境が連続的に変化する 2D 空間に分布する祖先種に何が起きるかを調査することは、興味深い将来の研究課題だ。おそらく、遺伝子流動のもとでも局所適応

が生じ、個体の分散の範囲が狭まる進化が起きると思われる。近隣集団間の遺伝子流動のため、ふつうは種分化は起きないが、もし、2D 空間内に弱い物理的障壁が存在すれば、それが強化され、発端種が生じる可能性があるだろう。実際、アマゾンの鳥類については、必ずしもそこで種分化が起きたとは限らないものの、通常なら障壁として機能しそうにない狭い川を境にして、多数の種が近縁種へと置き換わる現象が観察されている[54]。

2 つ目のモデル拡張の方向性は、島や環境の数をそれぞれ 4 つ以上に増やすことである。適応放散の実際の例は、通常、このような複雑な条件下で起きている。直感的にはそのような場合でも同期のメカニズムは依然として機能すると思われるが、この拡張を実際に行って確認することが重要だろう。しかし、この拡張には乗り越えるべき 2 つの障害が伴っている。

第一の障害は、移住先の選択確率の問題だ。オリジナルモデルはこの確率を、島間の近さ指標、つまり地理的距離の逆数に基づいて定義した。しかし、ある一定の範囲内に多数の島を配置していけば、いずれは非常に近接した島のペアが生じざるを得ない。そのような島ペアの近さ指標は非常に高くなるため、これらの島に住む祖先種が他の島に移動することは事実上不可能になってしまう。これは、高い分散力を持つ祖先種が小さな群島内のどこにでも簡単に移動できるという仮定と矛盾する。この問題を克服するには、移動先の選定確率を一般化して、島間の近さ指標から独立したものにすることが効果的だろう。

もう一つの障害は、種数判定の複雑さだ。オリジナルモデルは 3 島しか含まないため、3 つの平均移民数をモニタリングすることでこれを簡単に行えた。2 つの平均が OMPG 基準を満たしたときに最初の種分化が起き、3 つすべてが OMPG 基準を満たしたときに 2 回目の種分化が起きたと結論することができたのである。しかし、島数が増えると、そのような単純な判定方法の適用は難しくなる。島の組み合わせ数が爆発的に増えるうえ、各島に 1 種という最終状態に到達するまでのあいだに、さまざまな分布を持つ多様な種が生じるからだ。この問題の解決のためには、single-linkage（friends-of-friends）のクラスター分析を用いることができる。平均移民数を類似度とみなしてこの分析を行えば、1 個体以上

の移住でつながるクラスターを 1 つの種として正しく認識できる。

私たちは第二の方向性の多島多環境の拡張を実際に行い、10 島 5 環境のケースを扱うコードを開発した（補足コード 1 参照）。移住先の島の選定確率を等しく設定したうえで、20 回のシミュレーションを実施したところ、常に約 20〜30 世代という短い期間内に種数が 1 種から 10 種へと爆発的に増加する現象が確認された（補足図 1）。

ここで、種分化が起きた後、次の種分化が起きるまでにかかった世代数を種分化間隔とみなし、種分化間隔が 10 世代以下であるものを同時種分化と定義すると、同時種分化の頻度は 90%、種分化間隔最小値は 0、中央値は 2、最大値は 18 であった。どのシミュレーションでも 9 種目が生み出されるまでは種分化間隔が短く、同時種分化が頻発する一方（同時種分化頻度 100%、種分化間隔最小値 0、中央値 1、最大値 8）、理由は不明だが、9 種目が生じた後、10 種目が生じるまでには長い時間がかかった（同時種分化頻度 20%、種分化間隔最小値 2、中央値 13、最大値 18）。移住先の島の選択確率の設定に違いがあるため、あまり適切な比較ではないかもしれないが、9 種目までのパターンは島数が 3 のモデルの結果によく似ている（同時種分化の頻度 100%、種分化間隔最小値 0、中央値 1、最大値 5）。拡張モデルの挙動の詳細を理解することは、今後の重要な研究課題である。

補足説明 4｜日本語版の原稿

…

補足コード 1｜シミュレーションに使用した SAS と R のコード

…

Supplementary Code 1 | The SAS and R codes used for the simulations

(1) The SAS code for three-island simulations

```
/* parameter settings */
%let repeat=20; * the number of simulations run with the following settings;
%let step=100000; * the arbitrarily selected upper bound of generations;
%let num_migration_loci=1;
%let unfit=0.3; *unfit=1-divergent selection (DS);
%let mutation_rate=0.0001;
%let dist12=0.5; * the distances among islands A and B;
%let dist13=0.5; * the distances among islands A and C;
%let KA=100; %let KB=100; %let KC=100;
*population sizes (KA=NA, KB=NB, KC=NC);

proc iml;
KA=&KA; KB=&KB; KC=&KC;
SUM=KA+KB+KC;
log_Gmin=j(1,1,.); * for recording generations of the first speciation events;
log_lag=j(1,1,.); * for recording speciation intervals;

DO REPEAT=1 to &REPEAT;

/* first generation */
birth_island=j(KA,1,1)//j(KB,1,2)//j(KC,1,3);
num_sedentary_genes=j(SUM,1,0); * the summed numbers of derived (sedentary) alleles at loci controlling migration;
morphology=j(SUM,1,0);
current_island=j(SUM,1,.);
current_generation=birth_island||num_sedentary_genes||morphology||current_island;
current_migration_genes=j(SUM,&num_migration_loci,0);
gmin=0;
gmax=0;
calc_MPG_50generations_mean=j(50,3,.);
/* first generation having derived alleles already (only for diploids and q=1);
num_derived_alleles=60; * the maximum is SUM, not 2*SUM, because we assumed recessive derived alleles causing loss of dispersal ability always invade the archipelago as heterozygotes;
birth_island=j(KA,1,1)//j(KB,1,2)//j(KC,1,3);
```

```
current_island=j(SUM,1,.);
migrgenes=j(num_derived_alleles,1,1)//j(SUM-num_derived_alleles,1,0);
   tmp=j(SUM,1,.);
   call randgen(tmp,"Uniform");
   migrgenes=tmp||migrgenes;
   call sort(migrgenes,1);
   migrgenes=migrgenes[,2];
   current_migration_genes=j(SUM,1,0);
   tmp=j(1,1,.);
   do n=1 to SUM;
      if migrgenes[n]=1 then do;
         current_migration_genes[n]=1;
         call randgen(tmp,"Binomial",0.5,1);
         if tmp=1 then current_migration_genes[n]=2;
      end;
   end;
   num_sedentary_genes= j(SUM,1,0); *primitive allele dominance;
morphogenes=j(num_derived_alleles,1,1)//j(2*SUM-num_derived_alleles,1,0);
   tmp=j(2*SUM,1,.);
   call randgen(tmp,"Uniform");
   morphogenes=tmp||morphogenes;
   call sort(morphogenes,1);
   morphogenes=morphogenes[,2];
   morphogenes
      =morphogenes[1:SUM,]||morphogenes[SUM+1:2*SUM,];
   morphology=j(SUM,1,0);
   do n=1 to SUM;
      if morphogenes[n,]={0 1} then morphology[n]=1;
      if morphogenes[n,]={1 0} then morphology[n]=2;
      if morphogenes[n,]={1 1} then morphology[n]=3;
   end;
current_generation=birth_island||num_sedentary_genes||morphology||current_island;
gmin=0;
gmax=0;
calc_MPG_50generations_mean=j(50,3,.);
*/
```

```
DO STEP=1 TO &STEP;
if gmax>0 then goto ABORT;

/* migration */
dist12=&dist12; dist13=&dist13; dist23=1;
dist21=dist12; dist31=dist13; dist32=dist23;
prob_migration=(0.5/&num_migration_loci)*
(j(SUM,1,&num_migration_loci)-current_generation[,2]); * M=0.5;
/* for diploids;
prob_migration=(0.5/(2*&num_migration_loci))*
(j(SUM,1,2*&num_migration_loci)-current_generation[,2]);
*/
migr_or_not=j(SUM,1,.);
do n=1 to SUM;
   migr_or_not[n]=rand('BINOMIAL',prob_migration[n],1);
end;
/* for memory usage reduction;
tmp_b=j(1,1,.);
migr_or_not=j(SUM,1,.);
do n=1 to SUM;
  call randgen(tmp_b,"Binomial",prob_migration[n],1);
  migr_or_not[n]=tmp_b;
end;
*/

sumdist=dist12+dist13;
prob12=dist13/sumdist;
destination1=j(KA,1,.);
migr_or_not1=migr_or_not[1:KA,];

sumdist=dist21+dist23;
prob21=dist23/sumdist;
destination2=j(KB,1,.);
migr_or_not2=migr_or_not[(KA+1):(KA+KB),];
```

```
sumdist=dist31+dist32;
prob31=dist32/sumdist;
destination3=j(KC,1,.);
migr_or_not3=migr_or_not[(KA+KB+1):SUM,];

do n=1 to KA;
  if migr_or_not1[n]=0 then destination1[n]=1;
  if migr_or_not1[n]=1 then
    destination1[n]=ifn(rand('binomial',prob12,1)=1,2,3);
end;
/* for memory usage reduction;
tmp_b=j(KA,1,.);
call randgen(tmp_b,"Binomial",prob12,1);
do n=1 to KA;
if migr_or_not1[n]=0 then destination1[n]=1;
if migr_or_not1[n]=1 then do;
  destination1[n]=ifn(tmp_b[n]=1,2,3);
end;
end;
*/

do n=1 to KB;
  if migr_or_not2[n]=0 then destination2[n]=2;
  if migr_or_not2[n]=1 then
    destination2[n]=ifn(rand('binomial',prob21,1)=1,1,3);
end;
/* for memory usage reduction;
tmp_b=j(KB,1,.);
call randgen(tmp_b,"Binomial",prob21,1);
do n=1 to KB;
if migr_or_not2[n]=0 then destination2[n]=2;
if migr_or_not2[n]=1 then do;
  destination2[n]=ifn(tmp_b[n]=1,1,3);
end;
end;
*/
```

```
do n=1 to KC;
   if migr_or_not3[n]=0 then destination3[n]=3;
   if migr_or_not3[n]=1 then
     destination3[n]=ifn(rand('binomial',prob31,1)=1,1,2);
end;
/* for memory usage reduction;
tmp_b=j(KC,1,.);
call randgen(tmp_b,"Binomial",prob31,1);
do n=1 to KC;
if migr_or_not3[n]=0 then destination3[n]=3;
if migr_or_not3[n]=1 then do;
   destination3[n]=ifn(tmp_b[n]=1,1,2);
end;
end;
*/

destination=destination1//destination2//destination3;
current_generation[,4]=destination;

/* preparing data matrices for island populations after migration */
tmp=current_generation||current_migration_genes;
call sort(tmp,4);
current_generation=tmp[,1:4];
current_migration_genes=tmp[,5:(4+&num_migration_loci)];
tmp1=current_generation[,4]=1;
tmp2=current_generation[,4]=2;
tmp3=current_generation[,4]=3;
num1=tmp1[+,];
num2=tmp2[+,];
num3=tmp3[+,];
island1=current_generation[1:num1,];
island2=current_generation[(num1+1):(num1+num2),];
island3=current_generation[(num1+num2+1):(num1+num2+num3),];
migration_genes1=current_migration_genes[1:num1,];
migration_genes2=current_migration_genes[(num1+1):(num1+num2),];
```

```
migration_genes3=current_migration_genes[(num1+num2+1):(num1+num2+num3),];

fitness1=&unfit*(island1[,3]=0)+1*(island1[,3]=1);
fitness2=1*(island2[,3]=0)+&unfit*(island2[,3]=1);
fitness3=1*(island3[,3]=0)+&unfit*(island3[,3]=1);

/* for three types of environments;
 fitness1=&unfit*(island1[,3]=0)+&unfit*(island1[,3]=1)+1*(island1[,3]=2);
 fitness2=1*(island2[,3]=0)+&unfit*(island2[,3]=1)+&unfit*(island2[,3]=2);
 fitness3=&unfit*(island3[,3]=0)+1*(island3[,3]=1)+&unfit*(island3[,3]=2);
*/

/* for the ancestral type adapted to island A;
 fitness1=1*(island1[,3]=0)+&unfit*(island1[,3]=1);
 fitness2=&unfit*(island2[,3]=0)+1*(island2[,3]=1);
 fitness3=&unfit*(island3[,3]=0)+1*(island3[,3]=1);
*/

/* for diploids;
 fitness1=&unfit*(island1[,3]=0)+1*(island1[,3]=1)+1*(island1[,3]=2)+1*(island1[,3]=3);
 fitness2=1*(island2[,3]=0)+&unfit*(island2[,3]=1)+&unfit*(island2[,3]=2)+&unfit*(island2[,3]
=3);
 fitness3=1*(island3[,3]=0)+&unfit*(island3[,3]=1)+&unfit*(island3[,3]=2)+&unfit*(island3[,3]
=3);
 * The first terms for homozygotes for primitive alleles;
 * The second and third terms for heterozygotes. In the cases of dominance, the coefficients are
equal to those of homozygotes of dominant alleles. In the case of codominance, the coefficients are
the averages of those of the two kinds of homozygotes);
 * The fourth terms for homozygotes for derived alleles;
*/

island1=island1||fitness1;
island2=island2||fitness2;
island3=island3||fitness3;

/* mating */
```

```
row_num1=j(nrow(island1),1,.);
do n=1 to nrow(island1);
   row_num1[n,]=n;
end;
male1=sample(row_num1,KA,"replace",fitness1);
male1=male1`;
female1=sample(row_num1,KA,"replace",fitness1);
female1=female1`;

row_num2=j(nrow(island2),1,.);
do n=1 to nrow(island2);
   row_num2[n,]=n;
end;
male2=sample(row_num2,KB,"replace",fitness2);
male2=male2`;
female2=sample(row_num2,KB,"replace",fitness2);
female2=female2`;

row_num3=j(nrow(island3),1,.);
do n=1 to nrow(island3);
   row_num3[n,]=n;
end;
male3=sample(row_num3,KC,"replace",fitness3);
male3=male3`;
female3=sample(row_num3,KC,"replace",fitness3);
female3=female3`;

/* counting the numbers of migrants */
male1_origin = j(KA,1,.);
female1_origin = j(KA,1,.);
male2_origin = j(KB,1,.);
female2_origin = j(KB,1,.);
male3_origin = j(KC,1,.);
female3_origin = j(KC,1,.);

do n=1 to KA;
```

```
   male1_origin[n] = island1[male1[n],1];
   female1_origin[n] = island1[female1[n],1];
end;
do n=1 to KB;
   male2_origin[n] = island2[male2[n],1];
   female2_origin[n] = island2[female2[n],1];
end;
do n=1 to KC;
   male3_origin[n] = island3[male3[n],1];
   female3_origin[n] = island3[female3[n],1];
end;

male_1to2 =      male2_origin=1;
female_1to2 = female2_origin=1;
male_2to1 =      male1_origin=2;
female_2to1 = female1_origin=2;
num_migration1vs2
=      male_1to2[+]
+ female_1to2[+]
+      male_2to1[+]
+ female_2to1[+];

male_1to3 =      male3_origin=1;
female_1to3 = female3_origin=1;
male_3to1 =      male1_origin=3;
female_3to1 = female1_origin=3;
num_migration1vs3
=      male_1to3[+]
+ female_1to3[+]
+      male_3to1[+]
+ female_3to1[+];

male_2to3 =      male3_origin=2;
female_2to3 = female3_origin=2;
male_3to2 =      male2_origin=3;
female_3to2 = female2_origin=3;
```

```
num_migration2vs3
=       male_2to3[+]
+ female_2to3[+]
+       male_3to2[+]
+ female_3to2[+];

current_MPG=num_migration1vs2||num_migration1vs3||num_migration2vs3;

/* timing of the first and second speciation events */
m=mod(STEP,50)+ifn(mod(STEP,50)=0,50,0);
calc_MPG_50generations_mean[m,]=current_MPG;
if STEP>=50 then do;
  current_MPG_50generations_mean
    = STEP ||    mean(calc_MPG_50generations_mean);
  if Gmin=0 then do;
    tmpG=current_MPG_50generations_mean[,1];
    tmp=current_MPG_50generations_mean[,2:4]<2;
    if tmp={1 1 1} then do; Gmin=tmpG; end;
    if tmp={1 1 0} then do; Gmin=tmpG; end;
    if tmp={1 0 1} then do; Gmin=tmpG; end;
    if tmp={0 1 1} then do; Gmin=tmpG; end;
  end;
  if Gmax=0 then do;
    tmpG=current_MPG_50generations_mean[,1];
    tmp=current_MPG_50generations_mean[,2:4]<2;
    tmp=tmp[+];
    if tmp=3 then Gmax=tmpG;
  end;
end;

/* mutation */
offsprings1=j(KA,&num_migration_loci+1,.);
offsprings2=j(KB,&num_migration_loci+1,.);
offsprings3=j(KC,&num_migration_loci+1,.);

do n=1 to KA;
```

```
do m=1 to &num_migration_loci;
  tmp_migration
  =ifn(rand('binomial',0.5,1)=0, migration_genes1[male1[n],m],
  migration_genes1[female1[n],m]);
  offsprings1[n,m]=tmp_migration+rand('binomial',&mutation_rate,1)
  *ifn(tmp_migration=0,1,-1);
end;
morphology =ifn(rand('binomial',0.5,1)=0,island1[male1[n],3],
island1[female1[n],3]);
mutated_morphology
=morphology+rand('binomial',&mutation_rate,1)*ifn(morphology=0,1,-1);
/* for memory usage reduction;
num_migration_loci=&num_migration_loci;
mutation_rate=&mutation_rate;

tmp_b=j(KA,num_migration_loci+1,.);
call randgen(tmp_b,"Binomial",mutation_rate,1);
do n=1 to KA;
do m=1 to num_migration_loci;
  tmp_migration
  =ifn(sample({1 0},1)=0, migration_genes1[male1[n],m], migration_genes1[female1[n],m]);
  offsprings1[n,m]=tmp_migration+tmp_b[n,m]*ifn(tmp_migration=0,1,-1);
end;
morphology =ifn(sample({1 0},1)=0,island1[male1[n],3],island1[female1[n],3]);
mutated_morphology
=morphology+tmp_b[n,num_migration_loci+1]*ifn(morphology=0,1,-1);
*/
/* for three types of environments;
  if rand('binomial',&mutation_rate,1)=0 then mutated_morphology=morphology;
  if rand('binomial',&mutation_rate,1)=1 then do;
    if morphology=0 then
      mutated_morphology= ifn(rand('binomial',0.5,1)=0,1,2);
    if morphology=1 then
      mutated_morphology= ifn(rand('binomial',0.5,1)=0,0,2);
    if morphology=2 then
      mutated_morphology= ifn(rand('binomial',0.5,1)=0,0,1);
```

```
    end;
  */
  offsprings1[n,&num_migration_loci+1]=mutated_morphology;
end;

/* for diploids;
do n=1 to KA;
  do m=1 to &num_migration_loci;
    tmp1=migration_genes1[male1[n],m];
      * tmp1: the genotype of the parent;
        * 0: the homozygote for primitive alleles;
        * 1, 2: the heterozygotes;
        * 3: the homozygote for derived alleles;
    if tmp1=0 then tmp2=0; * the allele inherited form the parent;
    if tmp1=1 then tmp2=ifn(rand('binomial',0.5,1)=0,0,1);
    if tmp1=2 then tmp2=ifn(rand('binomial',0.5,1)=0,0,1);
    if tmp1=3 then tmp2=1;
    tmp2=tmp2+rand('binomial',&mutation_rate,1)*ifn(tmp2=0,1,-1);
    m_gamete=tmp2;
    tmp1=migration_genes1[female1[n],m];
    if tmp1=0 then tmp2=0; * the allele inherited form the parent;
    if tmp1=1 then tmp2=ifn(rand('binomial',0.5,1)=0,0,1);
    if tmp1=2 then tmp2=ifn(rand('binomial',0.5,1)=0,0,1);
    if tmp1=3 then tmp2=1;
    tmp2=tmp2+rand('binomial',&mutation_rate,1)*ifn(tmp2=0,1,-1);
    f_gamete=tmp2;
    zygote=m_gamete||f_gamete;
    if zygote={0 0} then tmp_migration=0;
    if zygote={0 1} then tmp_migration=1;
    if zygote={1 0} then tmp_migration=2;
    if zygote={1 1} then tmp_migration=3;
    offsprings1[n,m]=tmp_migration;
  end;
  tmp1= island1[male1[n],3];
  if tmp1=0 then tmp2=0;
  if tmp1=1 then tmp2=ifn(rand('binomial',0.5,1)=0,0,1);
```

```
  if tmp1=2 then tmp2=ifn(rand('binomial',0.5,1)=0,0,1);
  if tmp1=3 then tmp2=1;
  tmp2=tmp2+rand('binomial',&mutation_rate,1)*ifn(tmp2=0,1,-1);
  m_gamete=tmp2;
  tmp1= island1[female1[n],3];
  if tmp1=0 then tmp2=0;
  if tmp1=1 then tmp2=ifn(rand('binomial',0.5,1)=0,0,1);
  if tmp1=2 then tmp2=ifn(rand('binomial',0.5,1)=0,0,1);
  if tmp1=3 then tmp2=1;
  tmp2=tmp2+rand('binomial',&mutation_rate,1)*ifn(tmp2=0,1,-1);
  f_gamete=tmp2;
  zygote=m_gamete||f_gamete;
  if zygote={0 0} then tmp_morphology=0;
  if zygote={0 1} then tmp_morphology=1;
  if zygote={1 0} then tmp_morphology=2;
  if zygote={1 1} then tmp_morphology=3;
  offsprings1[n,&num_migration_loci+1]=tmp_morphology;
end;
*/

do n=1 to KB;
  do m=1 to &num_migration_loci;
    tmp_migration
    =ifn(rand('binomial',0.5,1)=0, migration_genes2[male2[n],m],
    migration_genes2[female2[n],m]);
    offsprings2[n,m]=tmp_migration+rand('binomial',&mutation_rate,1)
    *ifn(tmp_migration=0,1,-1);
  end;
  morphology =ifn(rand('binomial',0.5,1)=0,island2[male2[n],3],
  island2[female2[n],3]);
  mutated_morphology
  =morphology+rand('binomial',&mutation_rate,1)*ifn(morphology=0,1,-1);
  /* for memory usage reduction;
  tmp_b=j(KB,num_migration_loci+1,.);
  call randgen(tmp_b,"Binomial",mutation_rate,1);
  do n=1 to KB;
```

```
  do m=1 to num_migration_loci;
    tmp_migration
    =ifn(sample({1 0},1)=0, migration_genes2[male2[n],m], migration_genes2[female2[n],m]);
    offsprings2[n,m]=tmp_migration+tmp_b[n,m]*ifn(tmp_migration=0,1,-1);
  end;
  morphology =ifn(sample({1 0},1)=0,island2[male2[n],3],island2[female2[n],3]);
  mutated_morphology
  =morphology+tmp_b[n,num_migration_loci+1]*ifn(morphology=0,1,-1);
  */
  /* for three types of environments;
    if rand('binomial',&mutation_rate,1)=0 then mutated_morphology=morphology;
    if rand('binomial',&mutation_rate,1)=1 then do;
      if morphology=0 then
        mutated_morphology= ifn(rand('binomial',0.5,1)=0,1,2);
      if morphology=1 then
        mutated_morphology= ifn(rand('binomial',0.5,1)=0,0,2);
      if morphology=2 then
        mutated_morphology= ifn(rand('binomial',0.5,1)=0,0,1);
    end;
  */
  offsprings2[n,&num_migration_loci+1]=mutated_morphology;
end;

/* for diploids;
do n=1 to KB;
  do m=1 to &num_migration_loci;
    tmp1=migration_genes2[male2[n],m];
    if tmp1=0 then tmp2=0;
    if tmp1=1 then tmp2=ifn(rand('binomial',0.5,1)=0,0,1);
    if tmp1=2 then tmp2=ifn(rand('binomial',0.5,1)=0,0,1);
    if tmp1=3 then tmp2=1;
    tmp2=tmp2+rand('binomial',&mutation_rate,1)*ifn(tmp2=0,1,-1);
    m_gamete=tmp2;
    tmp1=migration_genes2[female2[n],m];
    if tmp1=0 then tmp2=0;
    if tmp1=1 then tmp2=ifn(rand('binomial',0.5,1)=0,0,1);
```

```
    if tmp1=2 then tmp2=ifn(rand('binomial',0.5,1)=0,0,1);
    if tmp1=3 then tmp2=1;
    tmp2=tmp2+rand('binomial',&mutation_rate,1)*ifn(tmp2=0,1,-1);
    f_gamete=tmp2;
    zygote=m_gamete||f_gamete;
    if zygote={0 0} then tmp_migration=0;
    if zygote={0 1} then tmp_migration=1;
    if zygote={1 0} then tmp_migration=2;
    if zygote={1 1} then tmp_migration=3;
    offsprings2[n,m]=tmp_migration;
  end;
  tmp1=island2[male2[n],3];
  if tmp1=0 then tmp2=0;
  if tmp1=1 then tmp2=ifn(rand('binomial',0.5,1)=0,0,1);
  if tmp1=2 then tmp2=ifn(rand('binomial',0.5,1)=0,0,1);
  if tmp1=3 then tmp2=1;
  tmp2=tmp2+rand('binomial',&mutation_rate,1)*ifn(tmp2=0,1,-1);
  m_gamete=tmp2;
  tmp1= island2[female2[n],3];
  if tmp1=0 then tmp2=0;
  if tmp1=1 then tmp2=ifn(rand('binomial',0.5,1)=0,0,1);
  if tmp1=2 then tmp2=ifn(rand('binomial',0.5,1)=0,0,1);
  if tmp1=3 then tmp2=1;
  tmp2=tmp2+rand('binomial',&mutation_rate,1)*ifn(tmp2=0,1,-1);
  f_gamete=tmp2;
  zygote=m_gamete||f_gamete;
  if zygote={0 0} then tmp_morphology=0;
  if zygote={0 1} then tmp_morphology=1;
  if zygote={1 0} then tmp_morphology=2;
  if zygote={1 1} then tmp_morphology=3;
  offsprings2[n,&num_migration_loci+1]=tmp_morphology;
end;
*/

do n=1 to KC;
  do m=1 to &num_migration_loci;
```

```
  tmp_migration
  =ifn(rand('binomial',0.5,1)=0, migration_genes3[male3[n],m],
  migration_genes3[female3[n],m]);
  offsprings3[n,m]=tmp_migration+rand('binomial',&mutation_rate,1)
  *ifn(tmp_migration=0,1,-1);
 end;
 morphology =ifn(rand('binomial',0.5,1)=0,island3[male3[n],3],
 island3[female3[n],3]);
 mutated_morphology
 =morphology+rand('binomial',&mutation_rate,1)*ifn(morphology=0,1,-1);
 /* for memory usage reduction;
 tmp_b=j(KC,num_migration_loci+1,.);
 call randgen(tmp_b,"Binomial",mutation_rate,1);
 do n=1 to KC;
 do m=1 to num_migration_loci;
  tmp_migration
  =ifn(sample({1 0},1)=0, migration_genes3[male3[n],m], migration_genes3[female3[n],m]);
  offsprings3[n,m]=tmp_migration+tmp_b[n,m]*ifn(tmp_migration=0,1,-1);
 end;
 morphology =ifn(sample({1 0},1)=0,island3[male3[n],3],island3[female3[n],3]);
 mutated_morphology
 =morphology+tmp_b[n,num_migration_loci+1]*ifn(morphology=0,1,-1);
 */
 /* for three types of environments;
  if rand('binomial',&mutation_rate,1)=0 then mutated_morphology=morphology;
  if rand('binomial',&mutation_rate,1)=1 then do;
   if morphology=0 then
    mutated_morphology= ifn(rand('binomial',0.5,1)=0,1,2);
   if morphology=1 then
    mutated_morphology= ifn(rand('binomial',0.5,1)=0,0,2);
   if morphology=2 then
    mutated_morphology= ifn(rand('binomial',0.5,1)=0,0,1);
  end;
 */
 offsprings3[n,&num_migration_loci+1]=mutated_morphology;
end;
```

```
/* for diploids;
do n=1 to KC;
 do m=1 to &num_migration_loci;
  tmp1=migration_genes3[male3[n],m];
  if tmp1=0 then tmp2=0;
  if tmp1=1 then tmp2=ifn(rand('binomial',0.5,1)=0,0,1);
  if tmp1=2 then tmp2=ifn(rand('binomial',0.5,1)=0,0,1);
  if tmp1=3 then tmp2=1;
  tmp2=tmp2+rand('binomial',&mutation_rate,1)*ifn(tmp2=0,1,-1);
  m_gamete=tmp2;
  tmp1=migration_genes3[female3[n],m];
  if tmp1=0 then tmp2=0;
  if tmp1=1 then tmp2=ifn(rand('binomial',0.5,1)=0,0,1);
  if tmp1=2 then tmp2=ifn(rand('binomial',0.5,1)=0,0,1);
  if tmp1=3 then tmp2=1;
  tmp2=tmp2+rand('binomial',&mutation_rate,1)*ifn(tmp2=0,1,-1);
  f_gamete=tmp2;
  zygote=m_gamete||f_gamete;
  if zygote={0 0} then tmp_migration=0;
  if zygote={0 1} then tmp_migration=1;
  if zygote={1 0} then tmp_migration=2;
  if zygote={1 1} then tmp_migration=3;
  offsprings3[n,m]=tmp_migration;
 end;
 tmp1=island3[male3[n],3];
 if tmp1=0 then tmp2=0;
 if tmp1=1 then tmp2=ifn(rand('binomial',0.5,1)=0,0,1);
 if tmp1=2 then tmp2=ifn(rand('binomial',0.5,1)=0,0,1);
 if tmp1=3 then tmp2=1;
 tmp2=tmp2+rand('binomial',&mutation_rate,1)*ifn(tmp2=0,1,-1);
 m_gamete=tmp2;
 tmp1=island3[female3[n],3];
 if tmp1=0 then tmp2=0;
 if tmp1=1 then tmp2=ifn(rand('binomial',0.5,1)=0,0,1);
 if tmp1=2 then tmp2=ifn(rand('binomial',0.5,1)=0,0,1);
```

```
  if tmp1=3 then tmp2=1;
  tmp2=tmp2+rand('binomial',&mutation_rate,1)*ifn(tmp2=0,1,-1);
  f_gamete=tmp2;
  zygote=m_gamete||f_gamete;
  if zygote={0 0} then tmp_morphology=0;
  if zygote={0 1} then tmp_morphology=1;
  if zygote={1 0} then tmp_morphology=2;
  if zygote={1 1} then tmp_morphology=3;
  offsprings3[n,&num_migration_loci+1]=tmp_morphology;
end;
*/

/* next generation */
current_migration_genes
=offsprings1[,1:&num_migration_loci]
//offsprings2[,1:&num_migration_loci]
//offsprings3[,1:&num_migration_loci];
birth_island=j(KA,1,1)//j(KB,1,2)//j(KC,1,3);
num_sedentary_genes=current_migration_genes[,+];
/* for diploids;
tmp=j(nrow(current_migration_genes),ncol(current_migration_genes),0);
do n=1 to nrow(current_migration_genes);
   do m=1 to ncol(current_migration_genes);
      if current_migration_genes[n,m]=1 then tmp[n,m]=2;
      if current_migration_genes[n,m]=2 then tmp[n,m]=2;
      if current_migration_genes[n,m]=3 then tmp[n,m]=2;
   end;
end;
num_sedentary_genes=tmp[,+];
* the numbers of effective stationary genes: they are zero and two in homozygotes of the primitive alleles and those of the derived alleles, respectively. They are zero (for dominance of the primitive alleles), one (for codominance), or two (for dominance of the derived alleles) in heterozygotes;
*/
morphology
=offsprings1[,&num_migration_loci+1]
//offsprings2[,&num_migration_loci+1]
```

```
   //offsprings3[,&num_migration_loci+1];
   current_island=j(SUM,1,.);
   current_generation=birth_island||num_sedentary_genes||morphology||current_island;

ABORT:
END;

lag=Gmax-Gmin; * the speciation interval;
log_Gmin=log_Gmin//Gmin;
log_lag=log_lag//lag;
END;

log_Gmin=log_Gmin[2:nrow(log_Gmin),];
log_lag=log_lag[2:nrow(log_lag),];
print log_Gmin log_lag;
```

(2) The SAS code for ten-island simulations

/* parameter settings */

```
   proc iml;
   MAX_STEP=10000; * the arbitrarily selected upper bound of generations;
   num_migration_loci=1;
   unfit=0.3; *unfit=1-divergent selection (DS);
   mutation_rate=0.0001;
   KA=100; KB=100; KC=100; KD=100; KE=100;
   KF=100; KG=100; KH=100; KI=100; KJ=100;
   *KA=NA, KB=NB, ... (population sizes);

   SUM=KA+KB+KC+KD+KE+KF+KG+KH+KI+KJ;
   log_MPG_50generations_mean=j(1,46,.);
```

/* first generation */

```
   birth_island=j(KA,1,1)//j(KB,1,2)//j(KC,1,3)//j(KD,1,4)//j(KE,1,5)//j(KF,1,6)//j(KG,1,7)//j(KH,1,
8)//j(KI,1,9)//j(KJ,1,10);
   num_sedentary_genes=j(SUM,1,0); * the summed numbers of derived (sedentary) alleles at loci
controlling migration;
   morphology=j(SUM,1,0);
```

```
current_island=j(SUM,1,.);
current_generation=birth_island||num_sedentary_genes||morphology||current_island;
current_migration_genes=j(SUM,num_migration_loci,0);
Gmax=0;
calc_MPG_50generations_mean=j(50,45,.);

DO STEP=1 TO MAX_STEP;
IF GMAX>0 THEN DO;
   IF STEP-GMAX>101 THEN GOTO ABORT;
END;

/* migration */
prob_migration=(0.5/num_migration_loci)*
(j(SUM,1,num_migration_loci)-current_generation[,2]); * M=0.5;

migr_or_not=j(SUM,1,.);
do n=1 to SUM;
migr_or_not[n]=rand('BINOMIAL',prob_migration[n],1);
end;

migr_or_not1=migr_or_not[1:KA,];
migr_or_not2=migr_or_not[(KA+1):(KA+KB),];
migr_or_not3=migr_or_not[(KA+KB+1):(KA+KB+KC),];
migr_or_not4=migr_or_not[(KA+KB+KC+1):(KA+KB+KC+KD),];
migr_or_not5=migr_or_not[(KA+KB+KC+KD+1):(KA+KB+KC+KD+KE),];
migr_or_not6=migr_or_not[(KA+KB+KC+KD+KE+1):(KA+KB+KC+KD+KE+KF),];
migr_or_not7=migr_or_not[(KA+KB+KC+KD+KE+KF+1):(KA+KB+KC+KD+KE+KF+KG),];
migr_or_not8=migr_or_not[(KA+KB+KC+KD+KE+KF+KG+1):(KA+KB+KC+KD+KE+KF+K
G+KH),];
migr_or_not9=migr_or_not[(KA+KB+KC+KD+KE+KF+KG+KH+1):(KA+KB+KC+KD+KE+K
F+KG+KH+KI),];
migr_or_not10=migr_or_not[(KA+KB+KC+KD+KE+KF+KG+KH+KI+1):(KA+KB+KC+KD+
KE+KF+KG+KH+KI+KJ),];

destination1=j(KA,1,.); destination2=j(KB,1,.); destination3=j(KC,1,.); destination4=j(KD,1,.);
destination5=j(KE,1,.);
```

```
destination6=j(KF,1,.); destination7=j(KG,1,.); destination8=j(KH,1,.); destination9=j(KI,1,.);
destination10=j(KJ,1,.);

do n=1 to KA;
if migr_or_not1[n]=0 then destination1[n]=1;
if migr_or_not1[n]=1 then destination1[n]=sample({2,3,4,5,6,7,8,9,10},1);
end;

do n=1 to KB;
if migr_or_not2[n]=0 then destination2[n]=2;
if migr_or_not2[n]=1 then destination2[n]=sample({1,3,4,5,6,7,8,9,10},1);
end;

do n=1 to KC;
if migr_or_not3[n]=0 then destination3[n]=3;
if migr_or_not3[n]=1 then destination3[n]=sample({1,2,4,5,6,7,8,9,10},1);
end;

do n=1 to KD;
if migr_or_not4[n]=0 then destination4[n]=4;
if migr_or_not4[n]=1 then destination4[n]=sample({1,2,3,5,6,7,8,9,10},1);
end;

do n=1 to KE;
if migr_or_not5[n]=0 then destination5[n]=5;
if migr_or_not5[n]=1 then destination5[n]=sample({1,2,3,4,6,7,8,9,10},1);
end;

do n=1 to KF;
if migr_or_not6[n]=0 then destination6[n]=6;
if migr_or_not6[n]=1 then destination6[n]=sample({1,2,3,4,5,7,8,9,10},1);
end;

do n=1 to KG;
if migr_or_not7[n]=0 then destination7[n]=7;
if migr_or_not7[n]=1 then destination7[n]=sample({1,2,3,4,5,6,8,9,10},1);
```

```
end;

do n=1 to KH;
if migr_or_not8[n]=0 then destination8[n]=8;
if migr_or_not8[n]=1 then destination8[n]=sample({1,2,3,4,5,6,7,9,10},1);
end;

do n=1 to KI;
if migr_or_not9[n]=0 then destination9[n]=9;
if migr_or_not9[n]=1 then destination9[n]=sample({1,2,3,4,5,6,7,8,10},1);
end;

do n=1 to KJ;
if migr_or_not10[n]=0 then destination10[n]=10;
if migr_or_not10[n]=1 then destination10[n]=sample({1,2,3,4,5,6,7,8,9},1);
end;
* when migrating, individuals are assumed to choose any island as their destination with equal
probability;

destination=
destination1//destination2//destination3//destination4//destination5//
destination6//destination7//destination8//destination9//destination10;
current_generation[,4]=destination;

/* preparing data matrices for island populations after migration */
tmp=current_generation||current_migration_genes;
call sort(tmp,4);
current_generation=tmp[,1:4];
current_migration_genes=tmp[,5:(4+num_migration_loci)];
tmp1=current_generation[,4]=1;
tmp2=current_generation[,4]=2;
tmp3=current_generation[,4]=3;
tmp4=current_generation[,4]=4;
tmp5=current_generation[,4]=5;
tmp6=current_generation[,4]=6;
tmp7=current_generation[,4]=7;
```

```
tmp8=current_generation[,4]=8;
tmp9=current_generation[,4]=9;
tmp10=current_generation[,4]=10;
num1=tmp1[+,];
num2=tmp2[+,];
num3=tmp3[+,];
num4=tmp4[+,];
num5=tmp5[+,];
num6=tmp6[+,];
num7=tmp7[+,];
num8=tmp8[+,];
num9=tmp9[+,];
num10=tmp10[+,];

island1=current_generation[1:num1,];
island2=current_generation[(num1+1):(num1+num2),];
island3=current_generation[(num1+num2+1):(num1+num2+num3),];
island4=current_generation[(num1+num2+num3+1):(num1+num2+num3+num4),];
island5=current_generation[(num1+num2+num3+num4+1):(num1+num2+num3+num4+num5),];
island6=current_generation[(num1+num2+num3+num4+num5+1):(num1+num2+num3+num4+n
um5+num6),];
island7=current_generation[(num1+num2+num3+num4+num5+num6+1):(num1+num2+num3+n
um4+num5+num6+num7),];
island8=current_generation[(num1+num2+num3+num4+num5+num6+num7+1):(num1+num2+n
um3+num4+num5+num6+num7+num8),];
island9=current_generation[(num1+num2+num3+num4+num5+num6+num7+num8+1):(num1+n
um2+num3+num4+num5+num6+num7+num8+num9),];
island10=current_generation[(num1+num2+num3+num4+num5+num6+num7+num8+num9+1):(
num1+num2+num3+num4+num5+num6+num7+num8+num9+num10),];
migration_genes1=current_migration_genes[1:num1,];
migration_genes2=current_migration_genes[(num1+1):(num1+num2),];
migration_genes3=current_migration_genes[(num1+num2+1):(num1+num2+num3),];
migration_genes4=current_migration_genes[(num1+num2+num3+1):(num1+num2+num3+num4
),];
migration_genes5=current_migration_genes[(num1+num2+num3+num4+1):(num1+num2+num3
+num4+num5),];
```

migration_genes6=current_migration_genes[(num1+num2+num3+num4+num5+1):(num1+num2+num3+num4+num5+num6),];

migration_genes7=current_migration_genes[(num1+num2+num3+num4+num5+num6+1):(num1+num2+num3+num4+num5+num6+num7),];

migration_genes8=current_migration_genes[(num1+num2+num3+num4+num5+num6+num7+1):(num1+num2+num3+num4+num5+num6+num7+num8),];

migration_genes9=current_migration_genes[(num1+num2+num3+num4+num5+num6+num7+num8+1):(num1+num2+num3+num4+num5+num6+num7+num8+num9),];

migration_genes10=current_migration_genes[(num1+num2+num3+num4+num5+num6+num7+num8+num9+1):(num1+num2+num3+num4+num5+num6+num7+num8+num9+num10),];

fitness1=unfit*(island1[,3]=0)+unfit*(island1[,3]=1)+unfit*(island1[,3]=2)+unfit*(island1[,3]=3)+1*(island1[,3]=4);

fitness2=unfit*(island2[,3]=0)+unfit*(island2[,3]=1)+unfit*(island2[,3]=2)+unfit*(island2[,3]=3)+1*(island2[,3]=4);

fitness3=unfit*(island3[,3]=0)+unfit*(island3[,3]=1)+unfit*(island3[,3]=2)+1*(island3[,3]=3)+unfit*(island3[,3]=4);

fitness4=unfit*(island4[,3]=0)+unfit*(island4[,3]=1)+unfit*(island4[,3]=2)+1*(island4[,3]=3)+unfit*(island4[,3]=4);

fitness5=unfit*(island5[,3]=0)+unfit*(island5[,3]=1)+1*(island5[,3]=2)+unfit*(island5[,3]=3)+unfit*(island5[,3]=4);

fitness6=unfit*(island6[,3]=0)+unfit*(island6[,3]=1)+1*(island6[,3]=2)+unfit*(island6[,3]=3)+unfit*(island6[,3]=4);

fitness7=unfit*(island7[,3]=0)+1*(island7[,3]=1)+unfit*(island7[,3]=2)+unfit*(island7[,3]=3)+unfit*(island7[,3]=4);

fitness8=unfit*(island8[,3]=0)+1*(island8[,3]=1)+unfit*(island8[,3]=2)+unfit*(island8[,3]=3)+unfit*(island8[,3]=4);

fitness9=1*(island9[,3]=0)+unfit*(island9[,3]=1)+unfit*(island9[,3]=2)+unfit*(island9[,3]=3)+unfit*(island9[,3]=4);

fitness10=1*(island10[,3]=0)+unfit*(island10[,3]=1)+unfit*(island10[,3]=2)+unfit*(island10[,3]=3)+unfit*(island10[,3]=4);

```
island1=island1||fitness1;
island2=island2||fitness2;
island3=island3||fitness3;
island4=island4||fitness4;
island5=island5||fitness5;
island6=island6||fitness6;
island7=island7||fitness7;
island8=island8||fitness8;
island9=island9||fitness9;
island10=island10||fitness10;

/* mating */
row_num1=j(nrow(island1),1,.);
do n=1 to nrow(island1);
   row_num1[n,]=n;
end;
male1=sample(row_num1,KA,"replace",fitness1);
male1=male1`;
female1=sample(row_num1,KA,"replace",fitness1);
female1=female1`;

row_num2=j(nrow(island2),1,.);
do n=1 to nrow(island2);
   row_num2[n,]=n;
end;
male2=sample(row_num2,KB,"replace",fitness2);
male2=male2`;
female2=sample(row_num2,KB,"replace",fitness2);
female2=female2`;

row_num3=j(nrow(island3),1,.);
do n=1 to nrow(island3);
   row_num3[n,]=n;
end;
male3=sample(row_num3,KC,"replace",fitness3);
male3=male3`;
```

```
female3=sample(row_num3,KC,"replace",fitness3);
female3=female3`;

row_num4=j(nrow(island4),1,.);
do n=1 to nrow(island4);
   row_num4[n,]=n;
end;
male4=sample(row_num4,KD,"replace",fitness4);
male4=male4`;
female4=sample(row_num4,KD,"replace",fitness4);
female4=female4`;

row_num5=j(nrow(island5),1,.);
do n=1 to nrow(island5);
   row_num5[n,]=n;
end;
male5=sample(row_num5,KE,"replace",fitness5);
male5=male5`;
female5=sample(row_num5,KE,"replace",fitness5);
female5=female5`;

row_num6=j(nrow(island6),1,.);
do n=1 to nrow(island6);
   row_num6[n,]=n;
end;
male6=sample(row_num6,KF,"replace",fitness6);
male6=male6`;
female6=sample(row_num6,KF,"replace",fitness6);
female6=female6`;

row_num7=j(nrow(island7),1,.);
do n=1 to nrow(island7);
   row_num7[n,]=n;
end;
male7=sample(row_num7,KG,"replace",fitness7);
male7=male7`;
```

```
female7=sample(row_num7,KG,"replace",fitness7);
female7=female7`;

row_num8=j(nrow(island8),1,.);
do n=1 to nrow(island8);
   row_num8[n,]=n;
end;
male8=sample(row_num8,KH,"replace",fitness8);
male8=male8`;
female8=sample(row_num8,KH,"replace",fitness8);
female8=female8`;

row_num9=j(nrow(island9),1,.);
do n=1 to nrow(island9);
   row_num9[n,]=n;
end;
male9=sample(row_num9,KI,"replace",fitness9);
male9=male9`;
female9=sample(row_num9,KI,"replace",fitness9);
female9=female9`;

row_num10=j(nrow(island10),1,.);
do n=1 to nrow(island10);
   row_num10[n,]=n;
end;
male10=sample(row_num10,KJ,"replace",fitness10);
male10=male10`;
female10=sample(row_num10,KJ,"replace",fitness10);
female10=female10`;

/* counting the numbers of migrants */
male1_origin = j(KA,1,.);
female1_origin = j(KA,1,.);
male2_origin = j(KB,1,.);
female2_origin = j(KB,1,.);
male3_origin = j(KC,1,.);
```

```
female3_origin = j(KC,1,.);
male4_origin = j(KD,1,.);
female4_origin = j(KD,1,.);
male5_origin = j(KE,1,.);
female5_origin = j(KE,1,.);
male6_origin = j(KF,1,.);
female6_origin = j(KF,1,.);
male7_origin = j(KG,1,.);
female7_origin = j(KG,1,.);
male8_origin = j(KH,1,.);
female8_origin = j(KH,1,.);
male9_origin = j(KI,1,.);
female9_origin = j(KI,1,.);
male10_origin = j(KJ,1,.);
female10_origin = j(KJ,1,.);

do n=1 to KA;
   male1_origin[n] = island1[male1[n],1];
   female1_origin[n] = island1[female1[n],1];
end;
do n=1 to KB;
   male2_origin[n] = island2[male2[n],1];
   female2_origin[n] = island2[female2[n],1];
end;
do n=1 to KC;
   male3_origin[n] = island3[male3[n],1];
   female3_origin[n] = island3[female3[n],1];
end;
do n=1 to KD;
   male4_origin[n] = island4[male4[n],1];
   female4_origin[n] = island4[female4[n],1];
end;
do n=1 to KE;
   male5_origin[n] = island5[male5[n],1];
   female5_origin[n] = island5[female5[n],1];
end;
```

```
do n=1 to KF;
   male6_origin[n] = island6[male6[n],1];
   female6_origin[n] = island6[female6[n],1];
end;
do n=1 to KG;
   male7_origin[n] = island7[male7[n],1];
   female7_origin[n] = island7[female7[n],1];
end;
do n=1 to KH;
   male8_origin[n] = island8[male8[n],1];
   female8_origin[n] = island8[female8[n],1];
end;
do n=1 to KI;
   male9_origin[n] = island9[male9[n],1];
   female9_origin[n] = island9[female9[n],1];
end;
do n=1 to KJ;
   male10_origin[n] = island10[male10[n],1];
   female10_origin[n] = island10[female10[n],1];
end;

male_1to2 =     male2_origin=1;
female_1to2 = female2_origin=1;
male_2to1 =     male1_origin=2;
female_2to1 = female1_origin=2;
num_migration1vs2
=     male_1to2[+]
+ female_1to2[+]
+     male_2to1[+]
+ female_2to1[+];
male_1to3 =     male3_origin=1;
female_1to3 = female3_origin=1;
male_3to1 =     male1_origin=3;
female_3to1 = female1_origin=3;
num_migration1vs3
=     male_1to3[+]
```

```
+ female_1to3[+]
+       male_3to1[+]
+ female_3to1[+];
male_1to4 =       male4_origin=1;
female_1to4 = female4_origin=1;
male_4to1 =       male1_origin=4;
female_4to1 = female1_origin=4;
num_migration1vs4
=       male_1to4[+]
+ female_1to4[+]
+       male_4to1[+]
+ female_4to1[+];
male_1to5 =       male5_origin=1;
female_1to5 = female5_origin=1;
male_5to1 =       male1_origin=5;
female_5to1 = female1_origin=5;
num_migration1vs5
=       male_1to5[+]
+ female_1to5[+]
+       male_5to1[+]
+ female_5to1[+];
male_1to6 =       male6_origin=1;
female_1to6 = female6_origin=1;
male_6to1 =       male1_origin=6;
female_6to1 = female1_origin=6;
num_migration1vs6
=       male_1to6[+]
+ female_1to6[+]
+       male_6to1[+]
+ female_6to1[+];
male_1to7 =       male7_origin=1;
female_1to7 = female7_origin=1;
male_7to1 =       male1_origin=7;
female_7to1 = female1_origin=7;
num_migration1vs7
=       male_1to7[+]
```

```
+ female_1to7[+]
+       male_7to1[+]
+ female_7to1[+];
male_1to8 =       male8_origin=1;
female_1to8 = female8_origin=1;
male_8to1 =       male1_origin=8;
female_8to1 = female1_origin=8;
num_migration1vs8
=       male_1to8[+]
+ female_1to8[+]
+       male_8to1[+]
+ female_8to1[+];
male_1to9 =       male9_origin=1;
female_1to9 = female9_origin=1;
male_9to1 =       male1_origin=9;
female_9to1 = female1_origin=9;
num_migration1vs9
=       male_1to9[+]
+ female_1to9[+]
+       male_9to1[+]
+ female_9to1[+];
male_1to10 =       male10_origin=1;
female_1to10 = female10_origin=1;
male_10to1 =       male1_origin=0;
female_10to1 = female1_origin=0;
num_migration1vs10
=       male_1to10[+]
+ female_1to10[+]
+       male_10to1[+]
+ female_10to1[+];

male_2to3 =       male3_origin=2;
female_2to3 = female3_origin=2;
male_3to2 =       male2_origin=3;
female_3to2 = female2_origin=3;
num_migration2vs3
```

```
=       male_2to3[+]
+ female_2to3[+]
+       male_3to2[+]
+ female_3to2[+];
male_2to4 =       male4_origin=2;
female_2to4 = female4_origin=2;
male_4to2 =       male2_origin=4;
female_4to2 = female2_origin=4;
num_migration2vs4
=       male_2to4[+]
+ female_2to4[+]
+       male_4to2[+]
+ female_4to2[+];
male_2to5 =       male5_origin=2;
female_2to5 = female5_origin=2;
male_5to2 =       male2_origin=5;
female_5to2 = female2_origin=5;
num_migration2vs5
=       male_2to5[+]
+ female_2to5[+]
+       male_5to2[+]
+ female_5to2[+];
male_2to6 =       male6_origin=2;
female_2to6 = female6_origin=2;
male_6to2 =       male2_origin=6;
female_6to2 = female2_origin=6;
num_migration2vs6
=       male_2to6[+]
+ female_2to6[+]
+       male_6to2[+]
+ female_6to2[+];
male_2to7 =       male7_origin=2;
female_2to7 = female7_origin=2;
male_7to2 =       male2_origin=7;
female_7to2 = female2_origin=7;
num_migration2vs7
```

```
=       male_2to7[+]
+ female_2to7[+]
+       male_7to2[+]
+ female_7to2[+];
male_2to8 =       male8_origin=2;
female_2to8 = female8_origin=2;
male_8to2 =       male2_origin=8;
female_8to2 = female2_origin=8;
num_migration2vs8
=       male_2to8[+]
+ female_2to8[+]
+       male_8to2[+]
+ female_8to2[+];
male_2to9 =       male9_origin=2;
female_2to9 = female9_origin=2;
male_9to2 =       male2_origin=9;
female_9to2 = female2_origin=9;
num_migration2vs9
=       male_2to9[+]
+ female_2to9[+]
+       male_9to2[+]
+ female_9to2[+];
male_2to10 =       male10_origin=2;
female_2to10 = female10_origin=2;
male_10to2 =       male2_origin=0;
female_10to2 = female2_origin=0;
num_migration2vs10
=       male_2to10[+]
+ female_2to10[+]
+       male_10to2[+]
+ female_10to2[+];

male_3to4 =       male4_origin=3;
female_3to4 = female4_origin=3;
male_4to3 =       male3_origin=4;
female_4to3 = female3_origin=4;
```

```
num_migration3vs4
=	male_3to4[+]
+ female_3to4[+]
+	male_4to3[+]
+ female_4to3[+];
male_3to5 =	male5_origin=3;
female_3to5 = female5_origin=3;
male_5to3 =	male3_origin=5;
female_5to3 = female3_origin=5;
num_migration3vs5
=	male_3to5[+]
+ female_3to5[+]
+	male_5to3[+]
+ female_5to3[+];
male_3to6 =	male6_origin=3;
female_3to6 = female6_origin=3;
male_6to3 =	male3_origin=6;
female_6to3 = female3_origin=6;
num_migration3vs6
=	male_3to6[+]
+ female_3to6[+]
+	male_6to3[+]
+ female_6to3[+];
male_3to7 =	male7_origin=3;
female_3to7 = female7_origin=3;
male_7to3 =	male3_origin=7;
female_7to3 = female3_origin=7;
num_migration3vs7
=	male_3to7[+]
+ female_3to7[+]
+	male_7to3[+]
+ female_7to3[+];
male_3to8 =	male8_origin=3;
female_3to8 = female8_origin=3;
male_8to3 =	male3_origin=8;
female_8to3 = female3_origin=8;
```

```
num_migration3vs8
=	male_3to8[+]
+ female_3to8[+]
+	male_8to3[+]
+ female_8to3[+];
male_3to9 =	male9_origin=3;
female_3to9 = female9_origin=3;
male_9to3 =	male3_origin=9;
female_9to3 = female3_origin=9;
num_migration3vs9
=	male_3to9[+]
+ female_3to9[+]
+	male_9to3[+]
+ female_9to3[+];
male_3to10 =	male10_origin=3;
female_3to10 = female10_origin=3;
male_10to3 =	male3_origin=0;
female_10to3 = female3_origin=0;
num_migration3vs10
=	male_3to10[+]
+ female_3to10[+]
+	male_10to3[+]
+ female_10to3[+];

male_4to5 =	male5_origin=4;
female_4to5 = female5_origin=4;
male_5to4 =	male4_origin=5;
female_5to4 = female4_origin=5;
num_migration4vs5
=	male_4to5[+]
+ female_4to5[+]
+	male_5to4[+]
+ female_5to4[+];
male_4to6 =	male6_origin=4;
female_4to6 = female6_origin=4;
male_6to4 =	male4_origin=6;
```

```
female_6to4 = female4_origin=6;
num_migration4vs6
=       male_4to6[+]
+ female_4to6[+]
+       male_6to4[+]
+ female_6to4[+];
male_4to7 =       male7_origin=4;
female_4to7 = female7_origin=4;
male_7to4 =       male4_origin=7;
female_7to4 = female4_origin=7;
num_migration4vs7
=       male_4to7[+]
+ female_4to7[+]
+       male_7to4[+]
+ female_7to4[+];
male_4to8 =       male8_origin=4;
female_4to8 = female8_origin=4;
male_8to4 =       male4_origin=8;
female_8to4 = female4_origin=8;
num_migration4vs8
=       male_4to8[+]
+ female_4to8[+]
+       male_8to4[+]
+ female_8to4[+];
male_4to9 =       male9_origin=4;
female_4to9 = female9_origin=4;
male_9to4 =       male4_origin=9;
female_9to4 = female4_origin=9;
num_migration4vs9
=       male_4to9[+]
+ female_4to9[+]
+       male_9to4[+]
+ female_9to4[+];
male_4to10 =       male10_origin=4;
female_4to10 = female10_origin=4;
male_10to4 =       male4_origin=0;
```

```
female_10to4 = female4_origin=0;
num_migration4vs10
=       male_4to10[+]
+ female_4to10[+]
+       male_10to4[+]
+ female_10to4[+];

male_5to6 =       male6_origin=5;
female_5to6 = female6_origin=5;
male_6to5 =       male5_origin=6;
female_6to5 = female5_origin=6;
num_migration5vs6
=       male_5to6[+]
+ female_5to6[+]
+       male_6to5[+]
+ female_6to5[+];
male_5to7 =       male7_origin=5;
female_5to7 = female7_origin=5;
male_7to5 =       male5_origin=7;
female_7to5 = female5_origin=7;
num_migration5vs7
=       male_5to7[+]
+ female_5to7[+]
+       male_7to5[+]
+ female_7to5[+];
male_5to8 =       male8_origin=5;
female_5to8 = female8_origin=5;
male_8to5 =       male5_origin=8;
female_8to5 = female5_origin=8;
num_migration5vs8
=       male_5to8[+]
+ female_5to8[+]
+       male_8to5[+]
+ female_8to5[+];
male_5to9 =       male9_origin=5;
female_5to9 = female9_origin=5;
```

```
male_9to5 =     male5_origin=9;
female_9to5 = female5_origin=9;
num_migration5vs9
=     male_5to9[+]
+ female_5to9[+]
+     male_9to5[+]
+ female_9to5[+];
male_5to10 =     male10_origin=5;
female_5to10 = female10_origin=5;
male_10to5 =     male5_origin=0;
female_10to5 = female5_origin=0;
num_migration5vs10
=     male_5to10[+]
+ female_5to10[+]
+     male_10to5[+]
+ female_10to5[+];

male_6to7 =     male7_origin=6;
female_6to7 = female7_origin=6;
male_7to6 =     male6_origin=7;
female_7to6 = female6_origin=7;
num_migration6vs7
=     male_6to7[+]
+ female_6to7[+]
+     male_7to6[+]
+ female_7to6[+];
male_6to8 =     male8_origin=6;
female_6to8 = female8_origin=6;
male_8to6 =     male6_origin=8;
female_8to6 = female6_origin=8;
num_migration6vs8
=     male_6to8[+]
+ female_6to8[+]
+     male_8to6[+]
+ female_8to6[+];
male_6to9 =     male9_origin=6;
```

```
female_6to9 = female9_origin=6;
male_9to6 =      male6_origin=9;
female_9to6 = female6_origin=9;
num_migration6vs9
=      male_6to9[+]
+ female_6to9[+]
+      male_9to6[+]
+ female_9to6[+];
male_6to10 =      male10_origin=6;
female_6to10 = female10_origin=6;
male_10to6 =      male6_origin=0;
female_10to6 = female6_origin=0;
num_migration6vs10
=      male_6to10[+]
+ female_6to10[+]
+      male_10to6[+]
+ female_10to6[+];

male_7to8 =      male8_origin=7;
female_7to8 = female8_origin=7;
male_8to7 =      male7_origin=8;
female_8to7 = female7_origin=8;
num_migration7vs8
=      male_7to8[+]
+ female_7to8[+]
+      male_8to7[+]
+ female_8to7[+];
male_7to9 =      male9_origin=7;
female_7to9 = female9_origin=7;
male_9to7 =      male7_origin=9;
female_9to7 = female7_origin=9;
num_migration7vs9
=      male_7to9[+]
+ female_7to9[+]
+      male_9to7[+]
+ female_9to7[+];
```

```
male_7to10 =        male10_origin=7;
female_7to10 = female10_origin=7;
male_10to7 =        male7_origin=0;
female_10to7 = female7_origin=0;
num_migration7vs10
=       male_7to10[+]
+ female_7to10[+]
+       male_10to7[+]
+ female_10to7[+];

male_8to9 =        male9_origin=8;
female_8to9 = female9_origin=8;
male_9to8 =        male8_origin=9;
female_9to8 = female8_origin=9;
num_migration8vs9
=       male_8to9[+]
+ female_8to9[+]
+       male_9to8[+]
+ female_9to8[+];
male_8to10 =        male10_origin=8;
female_8to10 = female10_origin=8;
male_10to8 =        male8_origin=0;
female_10to8 = female8_origin=0;
num_migration8vs10
=       male_8to10[+]
+ female_8to10[+]
+       male_10to8[+]
+ female_10to8[+];

male_9to10 =        male10_origin=9;
female_9to10 = female10_origin=9;
male_10to9 =        male9_origin=0;
female_10to9 = female9_origin=0;
num_migration9vs10
=       male_9to10[+]
+ female_9to10[+]
```

```
+       male_10to9[+]
+ female_10to9[+];

current_MPG=
num_migration1vs2||num_migration1vs3||num_migration1vs4||num_migration1vs5||num_migratio
n1vs6||
num_migration1vs7||num_migration1vs8||num_migration1vs9||num_migration1vs10||num_migrati
on2vs3||
num_migration2vs4||num_migration2vs5||num_migration2vs6||num_migration2vs7||num_migratio
n2vs8||
num_migration2vs9||num_migration2vs10||num_migration3vs4||num_migration3vs5||num_migrati
on3vs6||
num_migration3vs7||num_migration3vs8||num_migration3vs9||num_migration3vs10||num_migrati
on4vs5||
num_migration4vs6||num_migration4vs7||num_migration4vs8||num_migration4vs9||num_migratio
n4vs10||
num_migration5vs6||num_migration5vs7||num_migration5vs8||num_migration5vs9||num_migratio
n5vs10||
num_migration6vs7||num_migration6vs8||num_migration6vs9||num_migration6vs10||num_migrati
on7vs8||
num_migration7vs9||num_migration7vs10||num_migration8vs9||num_migration8vs10||num_migra
tion9vs10;

current_MPG=0.5*current_MPG;

/* timing of all island combinations meeting the OMPG rule */
m=mod(STEP,50)+ifn(mod(STEP,50)=0,50,0);
calc_MPG_50generations_mean[m,]=current_MPG;
if STEP>=50 then do;
   current_MPG_50generations_mean
   = STEP ||   mean(calc_MPG_50generations_mean);
   if Gmax=0 then do;
      tmpG=current_MPG_50generations_mean[,1];
      tmp=current_MPG_50generations_mean[,2:46]<1;
      tmp=tmp[+]; * counting elements that meet the OMPG rule;
      if tmp=45 then do; Gmax=tmpG; print Gmax; end; *45=10C2;
```

```
    end;
  end;

  log_MPG_50generations_mean=log_MPG_50generations_mean//current_MPG_50generations_m
ean;

  /* mutation */
  offsprings1=j(KA,num_migration_loci+1,.);
  offsprings2=j(KB,num_migration_loci+1,.);
  offsprings3=j(KC,num_migration_loci+1,.);
  offsprings4=j(KD,num_migration_loci+1,.);
  offsprings5=j(KE,num_migration_loci+1,.);
  offsprings6=j(KF,num_migration_loci+1,.);
  offsprings7=j(KG,num_migration_loci+1,.);
  offsprings8=j(KH,num_migration_loci+1,.);
  offsprings9=j(KI,num_migration_loci+1,.);
  offsprings10=j(KJ,num_migration_loci+1,.);

  do n=1 to KA;
    do m=1 to num_migration_loci;
      tmp_migration
      =ifn(rand('binomial',0.5,1)=0, migration_genes1[male1[n],m],
      migration_genes1[female1[n],m]);
      offsprings1[n,m]=tmp_migration+rand('binomial',mutation_rate,1)
      *ifn(tmp_migration=0,1,-1);
    end;
    morphology =ifn(rand('binomial',0.5,1)=0,island1[male1[n],3],
    island1[female1[n],3]);
    mutated_morphology=morphology;
    if rand('binomial',mutation_rate,1)= 1 then do;
      if morphology=0 then mutated_morphology=sample({1,2,3,4},1);
      if morphology=1 then mutated_morphology=sample({0,2,3,4},1);
      if morphology=2 then mutated_morphology=sample({0,1,3,4},1);
      if morphology=3 then mutated_morphology=sample({0,1,2,4},1);
      if morphology=4 then mutated_morphology=sample({0,1,2,3},1);
    end;
```

```
   offsprings1[n,num_migration_loci+1]=mutated_morphology;
end;

do n=1 to KB;
   do m=1 to num_migration_loci;
      tmp_migration
      =ifn(rand('binomial',0.5,1)=0, migration_genes2[male2[n],m],
      migration_genes2[female2[n],m]);
      offsprings2[n,m]=tmp_migration+rand('binomial',mutation_rate,1)
      *ifn(tmp_migration=0,1,-1);
   end;
   morphology =ifn(rand('binomial',0.5,1)=0,island2[male2[n],3],
   island2[female2[n],3]);
   mutated_morphology=morphology;
   if rand('binomial',mutation_rate,1)= 1 then do;
      if morphology=0 then mutated_morphology=sample({1,2,3,4},1);
      if morphology=1 then mutated_morphology=sample({0,2,3,4},1);
      if morphology=2 then mutated_morphology=sample({0,1,3,4},1);
      if morphology=3 then mutated_morphology=sample({0,1,2,4},1);
      if morphology=4 then mutated_morphology=sample({0,1,2,3},1);
   end;

   offsprings2[n,num_migration_loci+1]=mutated_morphology;
end;

do n=1 to KC;
   do m=1 to num_migration_loci;
      tmp_migration
      =ifn(rand('binomial',0.5,1)=0, migration_genes3[male3[n],m],
      migration_genes3[female3[n],m]);
      offsprings3[n,m]=tmp_migration+rand('binomial',mutation_rate,1)
      *ifn(tmp_migration=0,1,-1);
   end;
   morphology =ifn(rand('binomial',0.5,1)=0,island3[male3[n],3],
   island3[female3[n],3]);
```

```
    mutated_morphology=morphology;
    if rand('binomial',mutation_rate,1)= 1 then do;
        if morphology=0 then mutated_morphology=sample({1,2,3,4},1);
        if morphology=1 then mutated_morphology=sample({0,2,3,4},1);
        if morphology=2 then mutated_morphology=sample({0,1,3,4},1);
        if morphology=3 then mutated_morphology=sample({0,1,2,4},1);
        if morphology=4 then mutated_morphology=sample({0,1,2,3},1);
    end;

    offsprings3[n,num_migration_loci+1]=mutated_morphology;
end;

do n=1 to KD;
    do m=1 to num_migration_loci;
        tmp_migration
        =ifn(rand('binomial',0.5,1)=0, migration_genes4[male4[n],m],
        migration_genes4[female4[n],m]);
        offsprings4[n,m]=tmp_migration+rand('binomial',mutation_rate,1)
        *ifn(tmp_migration=0,1,-1);
    end;
    morphology =ifn(rand('binomial',0.5,1)=0,island4[male4[n],3],
    island4[female4[n],3]);
    mutated_morphology=morphology;
    if rand('binomial',mutation_rate,1)= 1 then do;
        if morphology=0 then mutated_morphology=sample({1,2,3,4},1);
        if morphology=1 then mutated_morphology=sample({0,2,3,4},1);
        if morphology=2 then mutated_morphology=sample({0,1,3,4},1);
        if morphology=3 then mutated_morphology=sample({0,1,2,4},1);
        if morphology=4 then mutated_morphology=sample({0,1,2,3},1);
    end;

    offsprings4[n,num_migration_loci+1]=mutated_morphology;
end;

do n=1 to KE;
    do m=1 to num_migration_loci;
```

```
        tmp_migration
        =ifn(rand('binomial',0.5,1)=0, migration_genes5[male5[n],m],
        migration_genes5[female5[n],m]);
        offsprings5[n,m]=tmp_migration+rand('binomial',mutation_rate,1)
        *ifn(tmp_migration=0,1,-1);
    end;
    morphology =ifn(rand('binomial',0.5,1)=0,island5[male5[n],3],
    island5[female5[n],3]);
    mutated_morphology=morphology;
    if rand('binomial',mutation_rate,1)= 1 then do;
        if morphology=0 then mutated_morphology=sample({1,2,3,4},1);
        if morphology=1 then mutated_morphology=sample({0,2,3,4},1);
        if morphology=2 then mutated_morphology=sample({0,1,3,4},1);
        if morphology=3 then mutated_morphology=sample({0,1,2,4},1);
        if morphology=4 then mutated_morphology=sample({0,1,2,3},1);
    end;

    offsprings5[n,num_migration_loci+1]=mutated_morphology;
end;

do n=1 to KF;
    do m=1 to num_migration_loci;
        tmp_migration
        =ifn(rand('binomial',0.5,1)=0, migration_genes6[male6[n],m],
        migration_genes6[female6[n],m]);
        offsprings6[n,m]=tmp_migration+rand('binomial',mutation_rate,1)
        *ifn(tmp_migration=0,1,-1);
    end;
    morphology =ifn(rand('binomial',0.5,1)=0,island6[male6[n],3],
    island6[female6[n],3]);
    mutated_morphology=morphology;
    if rand('binomial',mutation_rate,1)= 1 then do;
        if morphology=0 then mutated_morphology=sample({1,2,3,4},1);
        if morphology=1 then mutated_morphology=sample({0,2,3,4},1);
        if morphology=2 then mutated_morphology=sample({0,1,3,4},1);
        if morphology=3 then mutated_morphology=sample({0,1,2,4},1);
```

```
    if morphology=4 then mutated_morphology=sample({0,1,2,3},1);
  end;

  offsprings6[n,num_migration_loci+1]=mutated_morphology;
end;

do n=1 to KG;
  do m=1 to num_migration_loci;
    tmp_migration
    =ifn(rand('binomial',0.5,1)=0, migration_genes7[male7[n],m],
    migration_genes7[female7[n],m]);
    offsprings7[n,m]=tmp_migration+rand('binomial',mutation_rate,1)
    *ifn(tmp_migration=0,1,-1);
  end;
  morphology =ifn(rand('binomial',0.5,1)=0,island7[male7[n],3],
  island7[female7[n],3]);
  mutated_morphology=morphology;
  if rand('binomial',mutation_rate,1)= 1 then do;
    if morphology=0 then mutated_morphology=sample({1,2,3,4},1);
    if morphology=1 then mutated_morphology=sample({0,2,3,4},1);
    if morphology=2 then mutated_morphology=sample({0,1,3,4},1);
    if morphology=3 then mutated_morphology=sample({0,1,2,4},1);
    if morphology=4 then mutated_morphology=sample({0,1,2,3},1);
  end;

  offsprings7[n,num_migration_loci+1]=mutated_morphology;
end;

do n=1 to KH;
  do m=1 to num_migration_loci;
    tmp_migration
    =ifn(rand('binomial',0.5,1)=0, migration_genes8[male8[n],m],
    migration_genes8[female8[n],m]);
    offsprings8[n,m]=tmp_migration+rand('binomial',mutation_rate,1)
    *ifn(tmp_migration=0,1,-1);
  end;
```

```
    morphology =ifn(rand('binomial',0.5,1)=0,island8[male8[n],3],
    island8[female8[n],3]);
    mutated_morphology=morphology;
    if rand('binomial',mutation_rate,1)= 1 then do;
       if morphology=0 then mutated_morphology=sample({1,2,3,4},1);
       if morphology=1 then mutated_morphology=sample({0,2,3,4},1);
       if morphology=2 then mutated_morphology=sample({0,1,3,4},1);
       if morphology=3 then mutated_morphology=sample({0,1,2,4},1);
       if morphology=4 then mutated_morphology=sample({0,1,2,3},1);
    end;

    offsprings8[n,num_migration_loci+1]=mutated_morphology;
end;

do n=1 to KI;
    do m=1 to num_migration_loci;
       tmp_migration
       =ifn(rand('binomial',0.5,1)=0, migration_genes9[male9[n],m],
       migration_genes9[female9[n],m]);
       offsprings9[n,m]=tmp_migration+rand('binomial',mutation_rate,1)
       *ifn(tmp_migration=0,1,-1);
    end;
    morphology =ifn(rand('binomial',0.5,1)=0,island9[male9[n],3],
    island9[female9[n],3]);
    mutated_morphology=morphology;
    if rand('binomial',mutation_rate,1)= 1 then do;
       if morphology=0 then mutated_morphology=sample({1,2,3,4},1);
       if morphology=1 then mutated_morphology=sample({0,2,3,4},1);
       if morphology=2 then mutated_morphology=sample({0,1,3,4},1);
       if morphology=3 then mutated_morphology=sample({0,1,2,4},1);
       if morphology=4 then mutated_morphology=sample({0,1,2,3},1);
    end;

    offsprings9[n,num_migration_loci+1]=mutated_morphology;
end;
```

```
do n=1 to KJ;
   do m=1 to num_migration_loci;
      tmp_migration
      =ifn(rand('binomial',0.5,1)=0, migration_genes10[male10[n],m],
      migration_genes10[female10[n],m]);
      offsprings10[n,m]=tmp_migration+rand('binomial',mutation_rate,1)
      *ifn(tmp_migration=0,1,-1);
   end;
   morphology =ifn(rand('binomial',0.5,1)=0,island10[male10[n],3],
   island10[female10[n],3]);
   mutated_morphology=morphology;
   if rand('binomial',mutation_rate,1)= 1 then do;
      if morphology=0 then mutated_morphology=sample({1,2,3,4},1);
      if morphology=1 then mutated_morphology=sample({0,2,3,4},1);
      if morphology=2 then mutated_morphology=sample({0,1,3,4},1);
      if morphology=3 then mutated_morphology=sample({0,1,2,4},1);
      if morphology=4 then mutated_morphology=sample({0,1,2,3},1);
   end;

   offsprings10[n,num_migration_loci+1]=mutated_morphology;
end;

/* next generation */
current_migration_genes
= offsprings1[,1:num_migration_loci]
//offsprings2[,1:num_migration_loci]
//offsprings3[,1:num_migration_loci]
//offsprings4[,1:num_migration_loci]
//offsprings5[,1:num_migration_loci]
//offsprings6[,1:num_migration_loci]
//offsprings7[,1:num_migration_loci]
//offsprings8[,1:num_migration_loci]
//offsprings9[,1:num_migration_loci]
//offsprings10[,1:num_migration_loci];

birth_island=
```

```
j(KA,1,1)//j(KB,1,2)//j(KC,1,3)//j(KD,1,4)//j(KE,1,5)//
j(KF,1,6)//j(KG,1,7)//j(KH,1,8)//j(KI,1,9)//j(KJ,1,10);

num_sedentary_genes=current_migration_genes[,+];

morphology
= offsprings1[,num_migration_loci+1]
//offsprings2[,num_migration_loci+1]
//offsprings3[,num_migration_loci+1]
//offsprings4[,num_migration_loci+1]
//offsprings5[,num_migration_loci+1]
//offsprings6[,num_migration_loci+1]
//offsprings7[,num_migration_loci+1]
//offsprings8[,num_migration_loci+1]
//offsprings9[,num_migration_loci+1]
//offsprings10[,num_migration_loci+1];

current_island=j(SUM,1,.);
current_generation=birth_island||num_sedentary_genes||morphology||current_island;

ABORT:
END;

log_MPG_50generations_mean=log_MPG_50generations_mean[2:nrow(log_MPG_50generation
s_mean),];
print log_MPG_50generations_mean;
```

(3) The R code for calculating species numbers in ten-island simulations

```
# Store outputs from Code (2) except for the number of generations into the clipboard before
execution.
library("RRphylo")
MPG=read.table("clipboard",sep="¥t")
OUTPUT=matrix(nrow=nrow(MPG),ncol=1)

for (n in 1:nrow(MPG)){
  tmpMPG=as.matrix(MPG[n,])
```

```
tmp=as.numeric(tmpMPG<=1)
dist=matrix(nrow=10,ncol=10)
dist[1,2:10]=tmp[1:9]
dist[2,3:10]=tmp[10:17]
dist[3,4:10]=tmp[18:24]
dist[4,5:10]=tmp[25:30]
dist[5,6:10]=tmp[31:35]
dist[6,7:10]=tmp[36:39]
dist[7,8:10]=tmp[40:42]
dist[8,9:10]=tmp[43:44]
dist[9,10]=tmp[45]
dist[1,1]=0
dist[2,2]=0
dist[3,3]=0
dist[4,4]=0
dist[5,5]=0
dist[6,6]=0
dist[7,7]=0
dist[8,8]=0
dist[9,9]=0
dist[10,10]=0
dist[2:10,1]=tmp[1:9]
dist[3:10,2]=tmp[10:17]
dist[4:10,3]=tmp[18:24]
dist[5:10,4]=tmp[25:30]
dist[6:10,5]=tmp[31:35]
dist[7:10,6]=tmp[36:39]
dist[8:10,7]=tmp[40:42]
dist[9:10,8]=tmp[43:44]
dist[10,9]=tmp[45]
dist=as.dist(dist)

hc=hclust(dist,method="single")

if (sum(hc$height)==0){
  sp=1
```

```
  } else {
  sp=length(cutPhylo(as.phylo(hc),age=0)$tip.label)
  }

 OUTPUT[n,]=sp
 }

write.table(OUTPUT,"clipboard",row.names=FALSE,col.names=FALSE,sep="¥t")
```
